# Thrombectomy improves functional outcome in large core stroke partly by preventing severe cerebral edema: a real-world mediation analysis

**DOI:** 10.3389/fneur.2026.1936247

**Published:** 2026-10-16

**Authors:** Thien Quang Le, Son Van Dang Nguyen, Tuan Phuoc Pham, Tao Van Tran, Trung Quoc Nguyen, Alexander T. Yahanda, Adam A. Dmytriw, Vu Thanh Huynh, Hai Quang Duong, Trung Thanh Nguyen, Nhan Duc Le

**Affiliations:** 1 Stroke Department, Da Nang Hospital, Da Nang City, Vietnam; 2 Department of Cerebrovascular Disease, 115 People's Hospital, Ho Chi Minh City, Vietnam; 3 University of Medicine and Pharmacy at Ho Chi Minh City, Ho Chi Minh City, Vietnam; 4 Department of Neurosurgery, Washington University School of Medicine, St. Louis, MO, United States; 5 Oxford Neurovascular and Neuroradiology Unit, Nuffield Department of Surgical Sciences, Medical Sciences Division, University of Oxford, Oxford, United Kingdom; 6 Neuroendovascular Program, Massachusetts General Hospital & Brigham and Women's Hospital, Harvard Medical School, Boston, MA, United States; 7 Neurointerventional and Neuroanalytics Consortium (NAN-C), School of Medicine, Toronto Metropolitan University, Toronto, ON, Canada; 8 Radiology Department, Neurointervention Unit, Da Nang Hospital, Da Nang City, Vietnam; 9 Department of Intensive Care and Poisoning Control, Da Nang Hospital, Da Nang City, Vietnam; 10 The University of Danang - School of Medicine and Pharmacy, Da Nang City, Vietnam

**Keywords:** cerebral edema, endovascular treatment, large core, low ASPECTS, mass effect, stroke

## Abstract

**Objective:**

Endovascular thrombectomy (EVT) is effective in large infarct core (LIC) stroke, but mortality and severe disability remain high when cerebral edema (CED) develops. We assessed the effect of EVT on CED and outcomes in LIC patients within 24 h of stroke onset.

**Methods:**

In a prospective observational cohort study, we enrolled patients with large-vessel occlusion in the anterior circulation, National Institutes of Health Stroke Scale (NIHSS) score ≥ 6, Alberta Stroke Program Early CT Score (ASPECTS) ≤ 5, and admission within 24 h of onset. The primary outcome was mass effect (ME) due to severe cerebral edema, defined as a maximum midline shift (MLS) ≥ 5 mm on follow-up imaging within 72 h. The secondary outcome was favorable outcome, defined as modified Rankin Scale (mRS) score of 0–3 at 90 days. Safety outcomes were determined by death at 90 days, symptomatic intracranial hemorrhage (sICH) according to SITS-MOST criteria. Treatment effects were estimated using inverse probability of treatment weighting (IPTW) with doubly robust adjustment. Mediation analysis assessed whether ME contributed to the association between EVT and favorable outcome.

**Results:**

Of 202 patients (EVT group: 119; medical management group: 83), after IPTW adjustment, EVT was associated with lower odds of ME [31.1% vs. 51.8%; IPTW-adjusted odds ratio (aOR), 0.28; 95% CI, 0.12–0.67], higher odds of favorable outcome (aOR, 2.32; 95% CI, 1.01–5.31), and lower mortality (aOR 0.45; 95% CI, 0.21–0.97) with no excess sICH. ME independently predicted poor outcome (aOR for mRS 4–6, 7.7; 95% CI, 3.01–19.69), and approximately 29.5% of the association between EVT and favorable outcome was statistically mediated by ME attenuation. Two admission variables—lower baseline ASPECTS and higher admission glucose—independently predicted ME (optimism-corrected AUC 0.82).

**Conclusion:**

In large-core stroke, EVT was associated with better functional outcome and lower odds of mass effect. Approximately one-third of EVT's functional benefit was statistically mediated by attenuation of severe CED. ME independently predicted poor outcome, whereas lower ASPECTS and higher admission glucose identified patients at greater risk of severe swelling, supporting bedside risk stratification to guide intensified monitoring and edema-targeted management.

**Trial registration:**

ClinicalTrials.gov, Identifier [NCT06016348].

## Introduction

Acute ischemic stroke (AIS) from large vessel occlusion (LVO) is a leading cause of death and disability, and approximately 25% of patients rapidly progress to a large infarct core (LIC), resulting in very poor outcomes (1–3). In recent years, landmark randomized clinical trials (RCTs) have demonstrated the efficacy and safety of endovascular thrombectomy (EVT) in patients with large-core stroke (4–9), and contemporary guidelines now support EVT in appropriately selected cases (10). Nevertheless, the absolute rates of death and severe disability remain high despite EVT (11, 12), indicating that successful recanalization alone does not fully explain outcome heterogeneity and that secondary injury mechanisms remain critically important.

Among these mechanisms, cerebral edema (CED) is one of the most clinically consequential. In anterior circulation LVO, CED worsens quickly: over 1/3 of patients deteriorate within 24 h, 2/3 within 48 h, and mortality approaches 70% without early decompressive surgery (13–15). Several studies have shown that CED leading to midline shift (MLS), especially when MLS ≥ 5 mm with mass effect (ME), is a strong predictor of poor outcomes in AIS (16–18). Although EVT benefits patients with LIC, its impact on CED progression remains uncertain. Prior studies have reported seemingly inconsistent results: a pooled analysis of 7 RCTs and secondary analyses from ANGEL-ASPECT and SELECT2 suggested increased MLS after EVT in patients with very large cores (19–21), whereas other studies have indicated that reperfusion reduces the risk of CED, thereby improving functional outcomes (22–24). More recently, a multicenter registry analysis reported that successful reperfusion was associated with less malignant CED, particularly in patients with ASPECTS 3–5 (25). These conflicting findings indicate that the relationship between EVT and edema is complex and incompletely understood, highlighting the need for further investigation in LIC.

Importantly, the existing pooled and secondary analyses were derived mainly from rigorously selected trial populations treated in high-resource systems and were not designed to answer a question that is especially relevant to routine practice in low- and middle-income countries (LMICs): whether EVT performed under a simplified non-contrast computed tomography (NCCT) and computed tomography angiography (CTA) workflow changes the risk of early, severe CED compared with medical management (MM) alone. This distinction matters because, in LMICs, access to perfusion imaging, serial neuroimaging, thrombectomy, decompressive surgery, and neurocritical care may be constrained, all of which can influence both the detection and the consequences of edema. Although EVT has been proven to be feasible and beneficial in resource-limited settings (26), the development of cerebral edema in LIC leads to increased treatment costs, greater care burdens, and generally poor outcomes. Accordingly, an important evidence gap remains regarding the effect of EVT on severe CED and the factors that predict edema in this population. We therefore aimed, in patients with LIC presenting within 24 h and selected by a simplified NCCT/CTA protocol, to evaluate the effect of EVT vs. MM on severe cerebral edema and 90-day functional outcome; to determine whether attenuation of mass effect mediates the functional benefit of EVT, and to identify predictors of ME in this clinically challenging and resource-limited population.

## Materials and methods

### Study design and eligible patients

This was a prospective, single-center, observational study conducted from October 2023 to August 2025 at Da Nang Hospital, Vietnam, a comprehensive stroke center in an LMIC setting. The data for this analysis were derived from patients enrolled at our center as part of the multicenter PROMISE (Predictors of Good Outcomes in Thrombectomy for Large Infarct Core Stroke Evaluation) cohort (ClinicalTrials.gov Identifier: NCT06016348). The study protocol adhered to the Declaration of Helsinki and was approved by the Institutional Review Board of Da Nang Hospital (Institutional Review Board No. 2449/BVĐN-HĐYĐ). Written informed consent was obtained from all patients or their legal representatives. The study adhered to the Strengthening the Reporting of Observational Studies in Epidemiology (STROBE) guidelines.

Inclusion criteria were age ≥ 18 years; AIS due to LVO in the anterior circulation (internal carotid artery (ICA), M1 or proximal M2 segment of the middle cerebral artery (MCA), or tandem occlusion), confirmed by CTA; LIC, defined as an Alberta Stroke Program Early Computed Tomography Score (ASPECTS) ≤ 5 on NCCT at admission; moderate to severe neurological deficit, with an admission National Institutes of Health Stroke Scale (NIHSS) score ≥ 6; and presentation within 24 h of symptom onset. Exclusion criteria were prestroke disability [modified Rankin Scale (mRS) score ≥ 3]; intracranial hemorrhage identified by NCCT; multiple or bilateral ischemic strokes; acute vertebrobasilar occlusion; or severe end-stage medical conditions.

The primary outcome was the incidence of mass effect due to severe cerebral edema in the early stages, defined as a maximum MLS ≥ 5 mm on follow-up imaging within 72 h. Secondary outcomes included a favorable functional outcome (mRS score 0–3) and a shift in the mRS distribution at 90 days. Safety outcomes included symptomatic intracranial hemorrhage (sICH) according to the SITS-MOST criteria; any intracranial hemorrhage; all-cause mortality within 90 days; early neurological deterioration (END), defined as an increase in NIHSS score ≥ 4 points within 24 h; and decompressive craniectomy within 7 days. The 90-day follow-up mRS assessment was conducted by independent research coordinators, blinded to treatment assignment, via telephone or during outpatient visits.

### Imaging analysis and treatment protocol

Imaging data were evaluated by two independent ASPECTS-certified neurologists, and any disagreements were subsequently confirmed by an experienced radiologist. All assessors were blinded to clinical outcomes and treatment allocation. Follow-up imaging (NCCT or MRI) was routinely performed at 24 h (±6 h) after stroke onset in all patients, and repeat imaging within the first 72 h was obtained in cases of neurological deterioration, particularly decreased consciousness. In addition, in the EVT group, at least one additional NCCT within 72 h was mandatory according to protocol. Midline shift was measured in maximum millimeters at the level of the septum pellucidum on any follow-up imaging within 72 h. Measurements were performed by two independent radiologists blinded to the treatment allocation and clinical data; the average of the two measurements was used for analysis.

A two-step imaging protocol using NCCT and CTA was applied for patient selection. Recombinant tissue plasminogen activator (tPA) was considered for patients presenting within the 4.5-h time window, with administration determined at the discretion of the treating physician. EVT techniques were performed at the neurointerventionist's discretion. The degree of reperfusion was assessed using the modified Thrombolysis in Cerebral Infarction (mTICI) scale, with successful reperfusion defined as mTICI ≥ 2b (27).

### Statistical analysis

All statistical analyses were conducted using Stata version 17 (StataCorp LLC, College Station, TX, USA) and R version 4.5.3 (R Foundation for Statistical Computing, Vienna, Austria). Continuous variables were expressed as medians with interquartile ranges (IQR), while categorical variables were presented as frequencies and percentages. Baseline characteristics are presented descriptively, with between-group balance assessed using standardized mean differences rather than significance testing, as detailed below. For outcome comparisons, the Mann–Whitney *U* test was used for continuous variables and the Chi-square test or Fisher's exact test for categorical variables, as appropriate. A two-sided *p*-value < 0.05 was considered statistically significant for all tests.

To minimize selection bias inherent in the observational design, we employed Inverse Probability of Treatment Weighting (IPTW). Propensity scores were estimated using a multivariable logistic regression model, with covariates pre-specified based on clinical relevance and biological plausibility as known confounders of treatment selection and outcome, including age, sex, baseline NIHSS, ASPECTS, time from onset to admission, and occlusion site. Stabilized weights were calculated to reduce variance and improve the precision of treatment effect estimates. Covariate balance before and after weighting was assessed using standardized mean differences (SMD), with SMD < 0.1 considered indicative of optimal balance and SMD < 0.2 acceptable. To estimate treatment effects, we employed a doubly-robust estimation approach to account for both propensity-score weighting and any residual imbalance. Stroke etiology was retained in the outcome model as a clinically important confounder to provide doubly-robust adjustment.

Mediation analyses followed the counterfactual causal framework of Imai, Keele and Tingley (28) to evaluate whether attenuation of ME mediated the association between exposure and favorable outcome. Two parallel analyses were performed: (i) an intention-to-treat formulation with EVT vs. MM as the exposure, and (ii) an as-treated formulation with good (mTICI ≥ 2b) vs. poor reperfusion as the exposure. Mediator and outcome models were both logistic regressions adjusted for the same covariates as the propensity score model, with stroke etiology retained as an additional covariate. Inverse probability weights were not applied to these models; confounding control in the mediation analysis therefore rests on regression adjustment (Supplementary Section S2.2). The total treatment effect was decomposed into the average causal mediation effect (ACME) and the average direct effect (ADE); the proportion mediated was calculated as ACME/(ACME + ADE). Point estimates and 95% confidence intervals were obtained from 1,000 non-parametric bootstrap replicates using the percentile method.

To determine the independent association between EVT and the risk of ME, and between ME and poor functional outcome (mRS 4–6), weighted logistic regression models were fitted with additional adjustment for the covariates included in the propensity-score model, plus stroke etiology. Robust variance estimators were used to account for the weighted design. Results are reported as adjusted odds ratios (aOR) with 95% confidence intervals (CI). In a sensitivity analysis for the cerebral edema–outcome association, treatment was added to the outcome model to test the robustness of the estimate.

To identify independent predictors of ME, 10 pre-specified clinical and imaging variables were entered into a multivariable logistic regression (Supplementary Table S3A). A parsimonious model was derived by backward stepwise elimination using the Akaike Information Criterion (AIC); model performance was evaluated using the C-statistic (Area Under the Curve, AUC) and Hosmer–Lemeshow test, and internally validated via 500 bootstrap resamples to obtain optimism-corrected estimates.

### Subgroup analysis

Prespecified subgroup analyses were performed stratified by age ( ≤ 60 vs. >60 years), NIHSS score (< 20 vs. ≥20), ASPECTS (0–2 vs. 3–5), time window ( ≤ 6 h vs. >6 h), and collateral score (good vs. poor). For each subgroup, the treatment effect was estimated within IPTW-weighted strata, and heterogeneity of treatment effect was assessed using the interaction *p*-value between treatment and the stratification variable. Forest plots were generated to visualize subgroup-specific aORs with 95% CI.

We also compared the good reperfusion group (defined by patients in the EVT group with mTICI ≥ 2b) and the poor reperfusion group (defined as patients in the EVT group with mTICI < 2b and the MM group) to estimate the as-treated effect of successful recanalization on CED and functional outcomes.

## Results

### Patients characteristics

From October 2023 to August 2025, we screened 577 patients with AIS due to large vessel occlusions within 24 h of stroke onset. A total of 202 eligible patients with LIC were included in the analysis: 119 (58.9%) in the EVT group, and 83 (41.1%) in the MM group (Figure 1).

**Figure 1:**
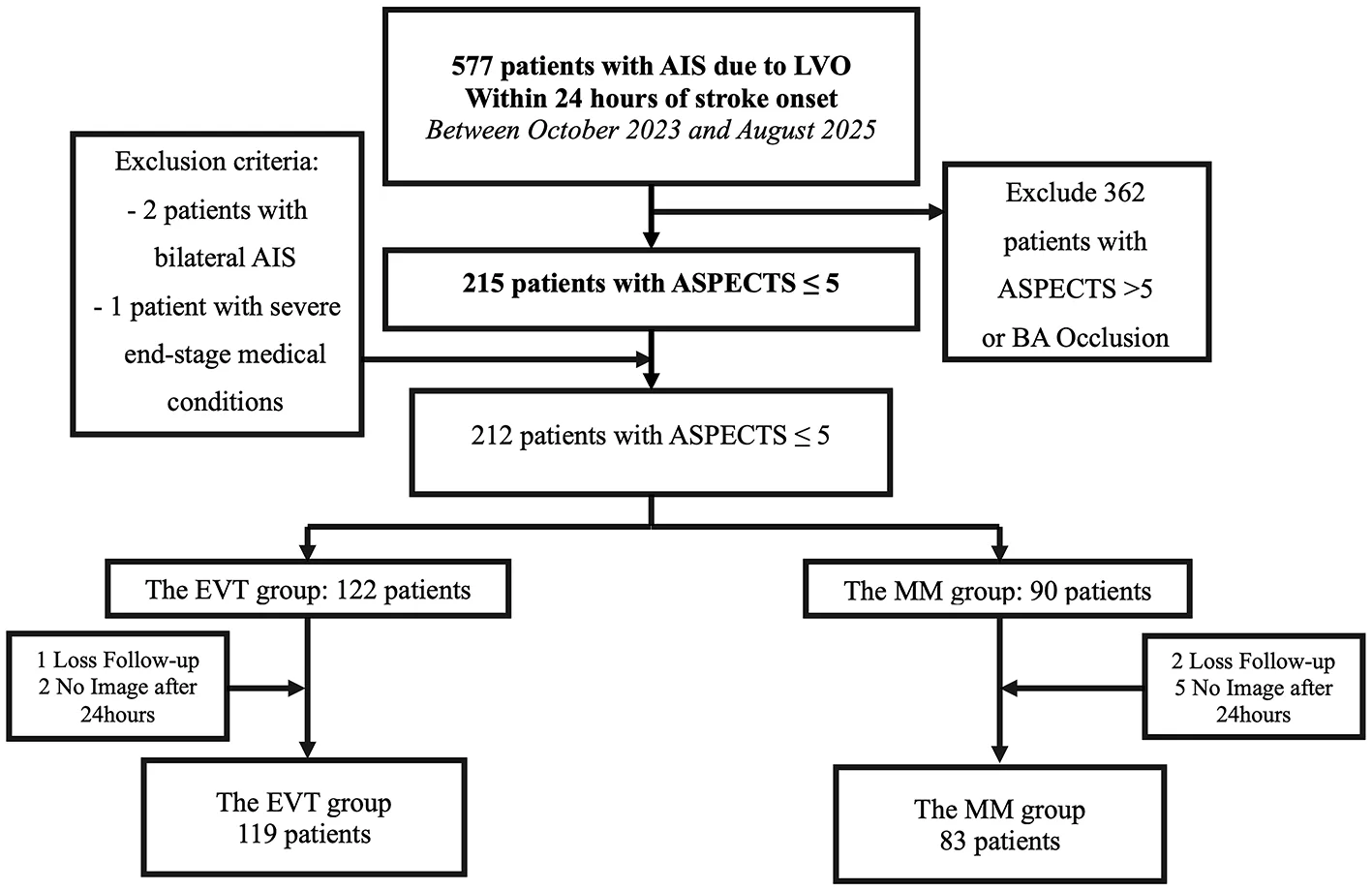
Study flow chart. Of 577 patients with anterior-circulation acute ischemic stroke due to large-vessel occlusion screened within 24 h of onset (October 2023–August 2025), 202 with large infarct core (ASPECTS ≤ 5) were included: 119 in the endovascular thrombectomy (EVT) group and 83 in the medical management (MM) group. AIS, acute ischemic stroke; ASPECTS, Alberta stroke program early CT score; BA, basilar artery; LVO, large-vessel occlusion.

In the overall cohort, significant baseline disparities were observed between the two treatment groups, indicating a strong selection bias. Specifically, patients in the EVT group were significantly younger (65 vs. 71 years; SMD 0.476), had lower NIHSS (17 vs. 18; SMD 0.281), presented with higher ASPECTS (4 vs. 3; SMD 0.415), and had a significantly shorter onset-to-admission time (3.5 vs. 6.9 h; SMD 0.611) compared to the MM group. Other baseline characteristics are detailed in Table 1 and Supplementary Table S1.

**Table 1:** Baseline characteristics and 90-day clinical outcomes before and after inverse probability of treatment weighting.

Characteristics of the patients	Overall cohort			IPTW cohort		
	EVT (119)	MM (83)	SMD	EVT (119)	MM (83)	SMD
**Age, years—Median (IQR)**	65 (59–74)	71 (64–83)	0.476	66 (60–78)	69 (59–78)	0.004
**Female sex—% (no.)**	50.4% (60)	43.4% (36)	0.142	45.5%	45.8%	0.007
**Pre-mRS—Median (IQR)**	0 (0–0)	0 (0–0)	0.115	0 (0–0)	0 (0–0)	0.138
**Medical History—% (no.)**						
TIA/Stroke	13.4% (16)	18.1% (15)	0.127	15.4%	19.7%	0.119
Hypertension	75.6% (90)	85.5% (71)	0.253	78.3%	83.5%	0.135
Atrial fibrillation	26.9% (32)	18.1% (15)	0.212	24.3%	16.2%	0.196
Diabetes mellitus	11.8% (14)	14.5% (12)	0.080	11.3%	12.6%	0.041
Dyslipidaemia	39.5% (47)	33.7% (28)	0.120	39.9%	34.7%	0.109
**Baseline NIHSS—Median (IQR)**	17 (14–21)	18 (16–21)	0.281	18 (14–22)	18 (15–20)	0.029
**Systolic blood pressure—Median (IQR)**	140 (120–150)	140 (130–160)	0.277	140 (120–160)	140 (120–160)	0.009
**Baseline ASPECTS—Median (IQR)**	4 (2–5)	3 (2–4)	0.415	4 (2–4)	4 (2–5)	0.016
0–2—no. (%)	25.2% (30)	41.0% (34)	0.340	27.8%	30.8%	0.065
3–5—no. (%)	74.8% (89)	59.0% (49)	0.340	72.2%	69.2%	0.065
**Occlusion site—no. (%)**						
ICA	6.7% (8)	14.5% (12)	0.253	8.1%	9.1%	0.031
M1 segment of MCA	47.1% (56)	53.0% (44)	0.119	51.6%	56.3%	0.093
M2 segment of MCA	0.8% (1)	2.4% (2)	0.124	1%	1.4%	0.029
Tandem	45.4% (54)	30.1% (25)	0.319	39.2%	33.3%	0.124
**Collateral score—Median (IQR)**	1 (1-2)	1 (1-2)	0.217	1 (1-2)	1 (1-2)	0.088
**Stroke etiology—no. (%)**						
Large-artery atherosclerosis	38.7% (46)	32.5% (27)	0.128	45.5%	29.7%	0.331
Cardioembolic	43.7% (52)	41.0% (34)	0.055	39.8%	39.1%	0.013
Undetermined or other	17.6% (21)	26.5% (22)	0.215	14.7%	31.2%	0.400
**Median time from onset to admission (IQR) (hours)**	3.5 (1.8–7.1)	6.9 (4.2–11.0)	0.611	4.8 (2.0–9.5)	4.9 (2.5–7.9)	0.008
≤ 6 h—no. (%)	70.6% (84)	45.8% (38)	0.520	60.4%	58.1%	0.048
>6 h—no. (%)	29.4% (35)	54.2% (45)		39.6%	41.9%	
**Outcomes**	**EVT (119)**	**MM (83)**	**cOR CI 95%**	***p***	**IPTW** + **aOR CI 95%**	***p***
**Primary outcomes**						
Mass effect—% (no.)	31.1% (37)	51.8% (43)	0.42 (0.24–0.75)	0.003	0.28 (0.12–0.67)	0.005
Midline shift (Median-IQR) (mm)	0 (0–8)	5 (0–10)	β: −1.91 (−3.77, −0.04)	0.047	β: −2.23 (−4.57, 0.12)	0.064
**Secondary outcome**						
Shift mRS at 90 days—Median (IQR)	4 (2–6)	6 (4–6)	0.34 (0.20–0.57)	< 0.001	0.48 (0.26–0.89)	0.020
**Score on the mRS at 90 days-% (no.)**						
mRS 0–2	25.2% (30)	4.8% (4)	6.66 (2.25–19.73)	< 0.001	4.40 (1.25–15.54)	0.022
mRS 0–3	47.1% (56)	19.3% (16)	3.72 (1.94–7.15)	< 0.001	2.32 (1.01–5.31)	0.048
**Safety outcomes**						
Symptomatic ICH—% (no.)	10.1% (12)	9.6% (8)	1.05 (0.41–2.70)	0.917	1.31 (0.43–3.94)	0.636
Any ICH—% (no.)	58.0% (69)	25.3% (21)	4.07 (2.20–7.53)	< 0.001	3.64 (1.66–7.96)	0.001
Death within 90 days—% (no.)	30.3% (36)	50.6% (42)	0.42 (0.24–0.76)	0.004	0.45 (0.21–0.97)	0.044
END % (no.)	38.7% (46)	57.8% (48)	0.46 (0.26–0.81)	0.008	0.42 (0.19–0.94)	0.035
Decompressive craniectomy within 7 days—% (no.)	7.6% (9)	8.4% (7)	0.89 (0.32–2.49)	0.822	1.54 (0.44–5.42)	0.502

Propensity score covariates (used to derive the IPTW weights): age, sex, baseline NIHSS, baseline ASPECTS, time from onset to admission, and occlusion site. Stroke etiology was not used in propensity score estimation but was retained in subsequent outcome models as a doubly-robust adjustment because its imbalance increased after weighting (SMD 0.128 to 0.331 for large-artery atherosclerosis). cOR was estimated by unweighted logistic regression. IPTW-aOR was estimated by weighted logistic regression with stabilized IPTW weights and additional doubly-robust covariate adjustment for age, sex, baseline NIHSS, baseline ASPECTS, time from onset to admission, occlusion site, and stroke etiology. β denotes the linear regression coefficient for continuous midline shift in millimeters.

aOR, adjusted odds ratio; ASPECTS, Alberta stroke program early CT Score; CI, confidence interval; cOR, crude odds ratio; END, early neurological deterioration (NIHSS increase ≥ 4 within 24 h); EVT, endovascular thrombectomy; ICA, internal carotid artery; ICH, intracranial hemorrhage; IPTW, inverse probability of treatment weighting; IQR, interquartile range; M1/M2, first/second segments of the middle cerebral artery; MCA, middle cerebral artery; MLS, midline shift; MM, medical management; mRS, modified Rankin Scale; NIHSS, National Institutes of Health Stroke Scale; sICH, symptomatic intracranial hemorrhage; SMD, standardized mean difference; TIA, transient ischemic attack.

After IPTW, these imbalances were substantially reduced. Age, sex, admission NIHSS, baseline ASPECTS and time from onset to arrival all achieved standardized mean differences below 0.03 (0.004, 0.007, 0.029, 0.016 and 0.008 respectively), and the largest of the four occlusion-site contrasts was 0.124. Fifteen of the 36 covariate contrasts fell below 0.10 and 16 lay between 0.10 and 0.20. Five remained above 0.20: the two stroke-etiology categories, intravenous thrombolysis, and two comorbidity variables reported in Supplementary Table S1. Stroke etiology, which was not among the propensity-score covariates, showed the largest increase in imbalance after weighting (large-artery atherosclerosis, SMD 0.128 to 0.331) and was therefore explicitly included as a covariate in the final doubly robust multivariable regression models; the remaining three are considered in the Limitations and in Supplementary Section S1.3.

### Primary outcome

Patients in the EVT group had a significantly lower incidence of ME than those in the MM group (31.1% vs. 51.8%). In the doubly robust IPTW analysis, EVT was associated with a reduced risk of developing mass effect (aOR = 0.28, 95% CI 0.12–0.67). Consistent with this finding, the median maximum MLS was notably less in the EVT group (0.0 mm, IQR 0–8) compared to the medical group (5.0 mm, IQR 0–10). Mass effect was strongly associated with poor functional outcome, increasing the odds of mRS 4–6 by more than seven-fold (IPTW-aOR 7.7; 95% CI, 3.01–19.69).

### Secondary and safety outcome

In the unadjusted analysis, the EVT group was associated with a higher rate of favorable outcome (47.1% vs. 19.3%, *p* < 0.001) and a lower rate of mortality at 90 days (30.3% vs. 50.6%, *p* = 0.004) and early neurological deterioration (38.7% vs. 57.8%, *p* = 0.008) compared with the MM group. In the doubly robust IPTW analysis, significant differences persisted, with the EVT group showing a higher rate of favorable outcome (aOR 2.32, 95% CI 1.01–5.31), lower 90-day mortality (aOR 0.45, 95% CI 0.21–0.97), and END (aOR 0.42, 95% CI 0.19–0.94). Although the EVT group had a higher rate of any ICH (aOR 3.64, 95% CI 1.66–7.96), the rate of sICH did not differ (aOR 1.31, 95% CI 0.43–3.94). The decompressive craniectomy rate between the two groups did not differ before or after IPTW analysis (Supplementary Table S4). The full distribution of 90-day mRS scores is shown in Figure 2, and the outcomes are detailed in Table 1.

**Figure 2:**
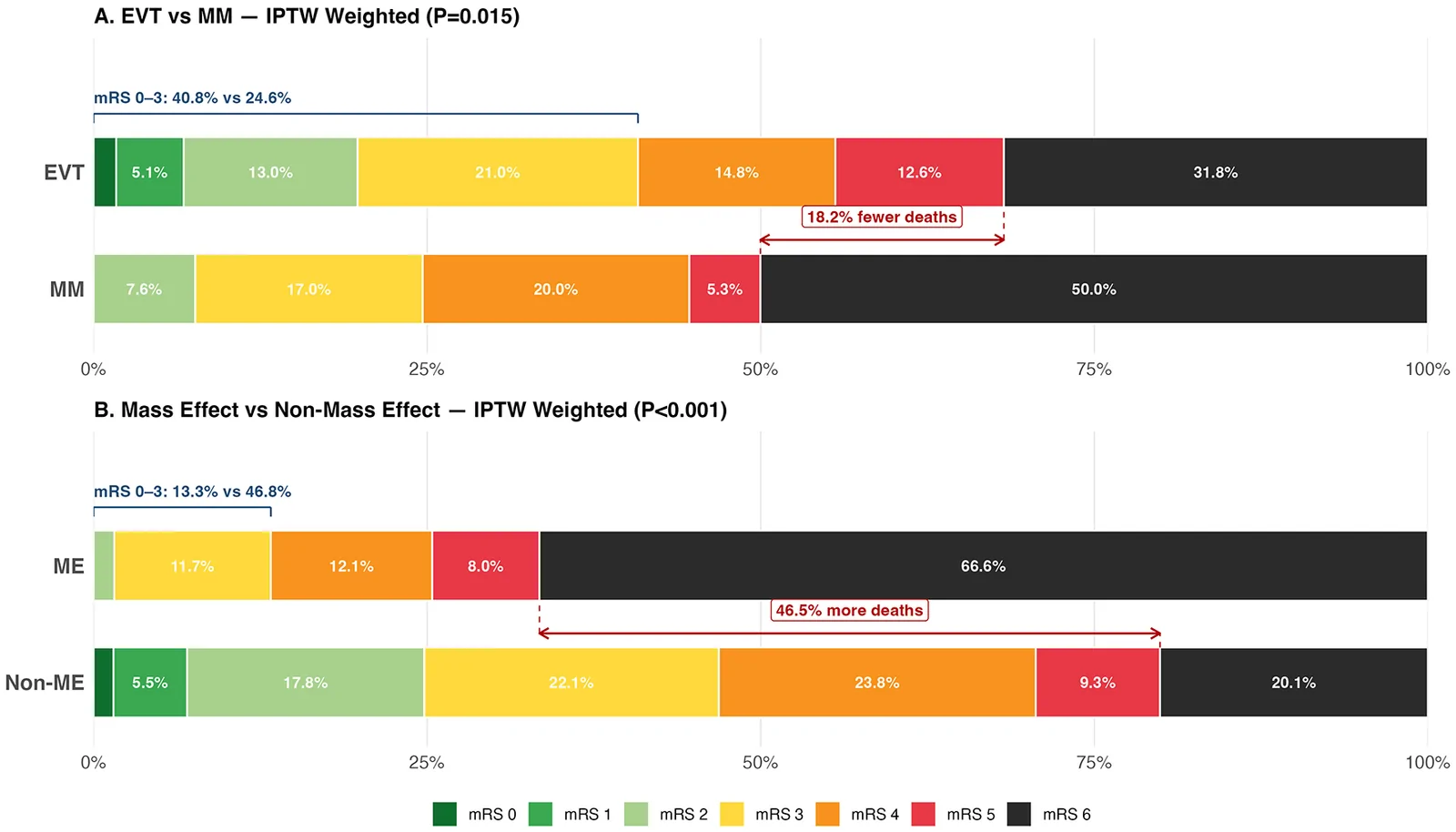
Distribution of 90-day functional outcomes by treatment group and by mass effect status, after IPTW adjustment. The stacked bar charts show the distribution of mRS scores ranging from 0 (no symptoms) to 6 (death). Both panels are IPTW-adjusted using stabilized weights derived from age, sex, baseline NIHSS, baseline ASPECTS, time from onset to admission, and occlusion site, with doubly-robust adjustment for stroke etiology. **(A)** Comparison of EVT vs. medical management (MM). **(B)** Comparison of patients who developed clinically significant mass effect (ME, defined as midline shift ≥ 5 mm on follow-up imaging within 72 h) vs. those who did not (Non-ME). Numbers within bars represent the percentage of patients in each mRS category. EVT, endovascular thrombectomy; IPTW, inverse probability of treatment weighting; ME, mass effect; MM, medical management; mRS, modified Rankin Scale.

### Mediation analysis

In mediation analyses, two parallel analyses were consistent with approximately 30% of the EVT benefit being statistically mediated by attenuation of ME at the favorable-outcome endpoint. In the intention-to-treat comparison (EVT vs. MM), 29.5% of the total effect was mediated through ME, although the indirect path did not reach statistical significance (ACME, 0.044; 95% CI, −0.005 to 0.107; total effect, 0.148; 95% CI, 0.003–0.293). In the as-treated comparison (good vs. poor reperfusion), 29.0% was mediated through ME, with a statistically significant indirect path (ACME, 0.060; 95% CI, 0.013–0.120; total effect, 0.206; 95% CI, 0.068–0.353) (Figure 3). The proportion mediated rose monotonically with mRS threshold laxity, reaching 41.5% at mRS ≤ 4 in the as-treated analysis (Supplementary Table S2).

**Figure 3:**
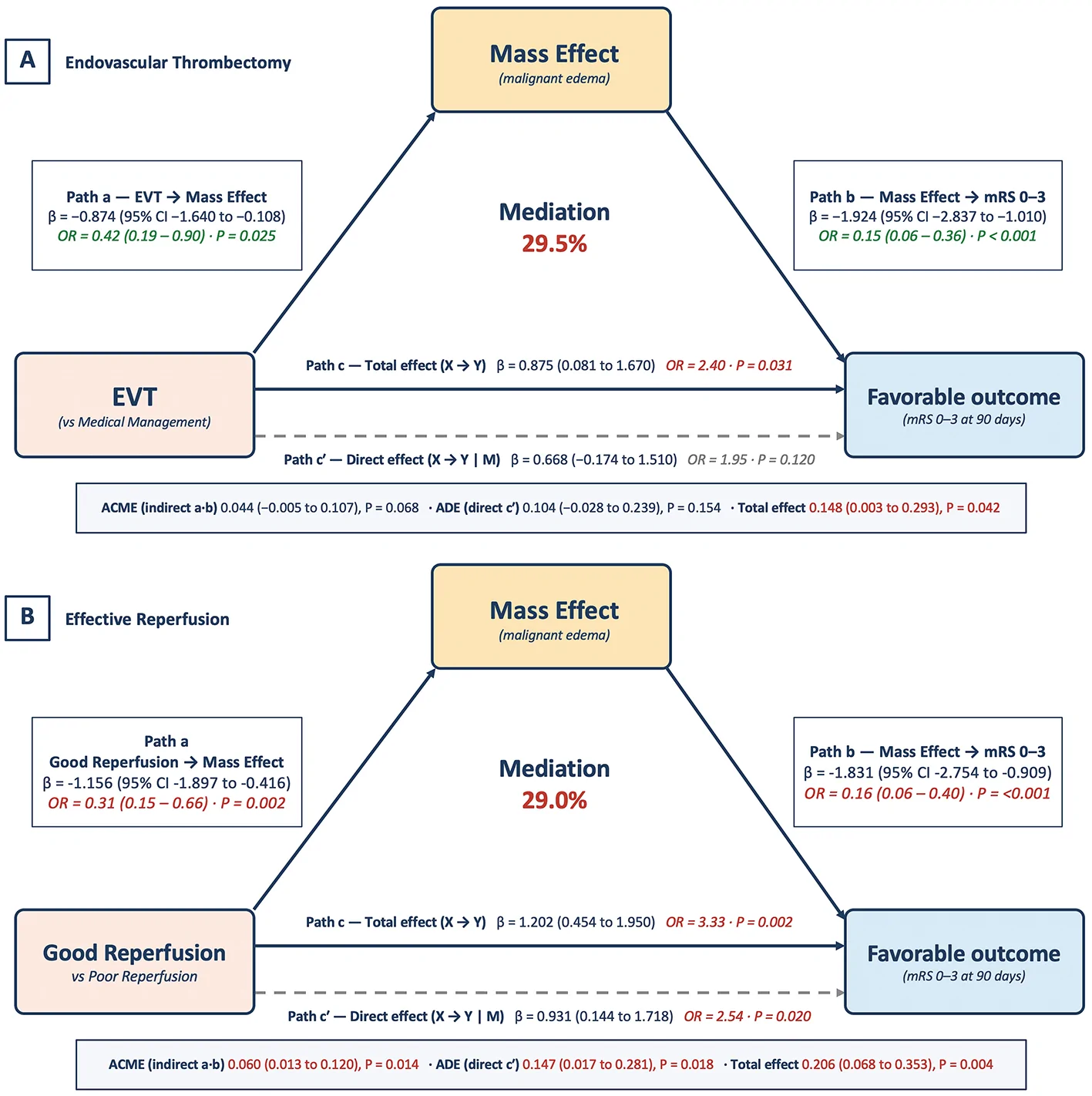
Causal mediation analysis: mass effect as a mediator of EVT and reperfusion benefit. Path diagrams from causal mediation analysis evaluating mass effect as a mediator of the association between exposure and 90-day favorable functional outcome (mRS 0–3). Both analyses used the counterfactual framework of Imai, Keele, and Tingley. Mediator and outcome models were logistic regressions adjusted for the propensity-score covariates and stroke etiology; inverse probability weights were not applied to these models (Supplementary Section S2.2). Point estimates and 95% confidence intervals were obtained from 1,000 nonparametric bootstrap replicates using the percentile method. For each panel, Path a represents the exposure → mediator association (effect of treatment/reperfusion on the development of mass effect); Path b represents the mediator → outcome association (effect of mass effect on favorable outcome) conditional on exposure; Path c represents the total effect of exposure on outcome; and Path c′ represents the direct effect of exposure on outcome after conditioning on mass effect. Path coefficients are shown as β with 95% CI (logit scale) and as the corresponding odds ratios (OR). The proportion mediated, displayed centrally, was calculated as ACME (ACME + ADE). **(A)** Intention-to-treat analysis comparing endovascular thrombectomy (EVT) versus medical management. **(B)** As-treated analysis comparing patients with good reperfusion (mTICI ≥ 2b) vs. poor reperfusion (mTICI < 2b in the EVT arm plus all medical-management patients). ACME, average causal mediation effect (indirect effect, a·b); ADE, average direct effect (c′); CI, confidence interval; EVT, endovascular thrombectomy; IPTW, inverse probability of treatment weighting; mRS, modified Rankin Scale; mTICI, modified Thrombolysis in Cerebral Infarction; OR, odds ratio; β, regression coefficient on the logit scale.

### Independent predictors of mass effect

In the final multivariable prognostic model, two predictors independently predicted ME: lower baseline ASPECTS (aOR, 0.45 per 1-point decrease; 95% CI, 0.34–0.59; *p* < 0.001) and higher admission plasma glucose (aOR, 1.02 per mg/dL; 95% CI, 1.01–1.02; *p* < 0.001) (Figure 4, Supplementary Table S3B). On 500-iteration non-parametric bootstrap internal validation, the parsimonious model showed excellent discrimination (optimism-corrected AUC, 0.82) and good calibration (Hosmer–Lemeshow *p* = 0.41; Brier score, 0.16).

**Figure 4:**
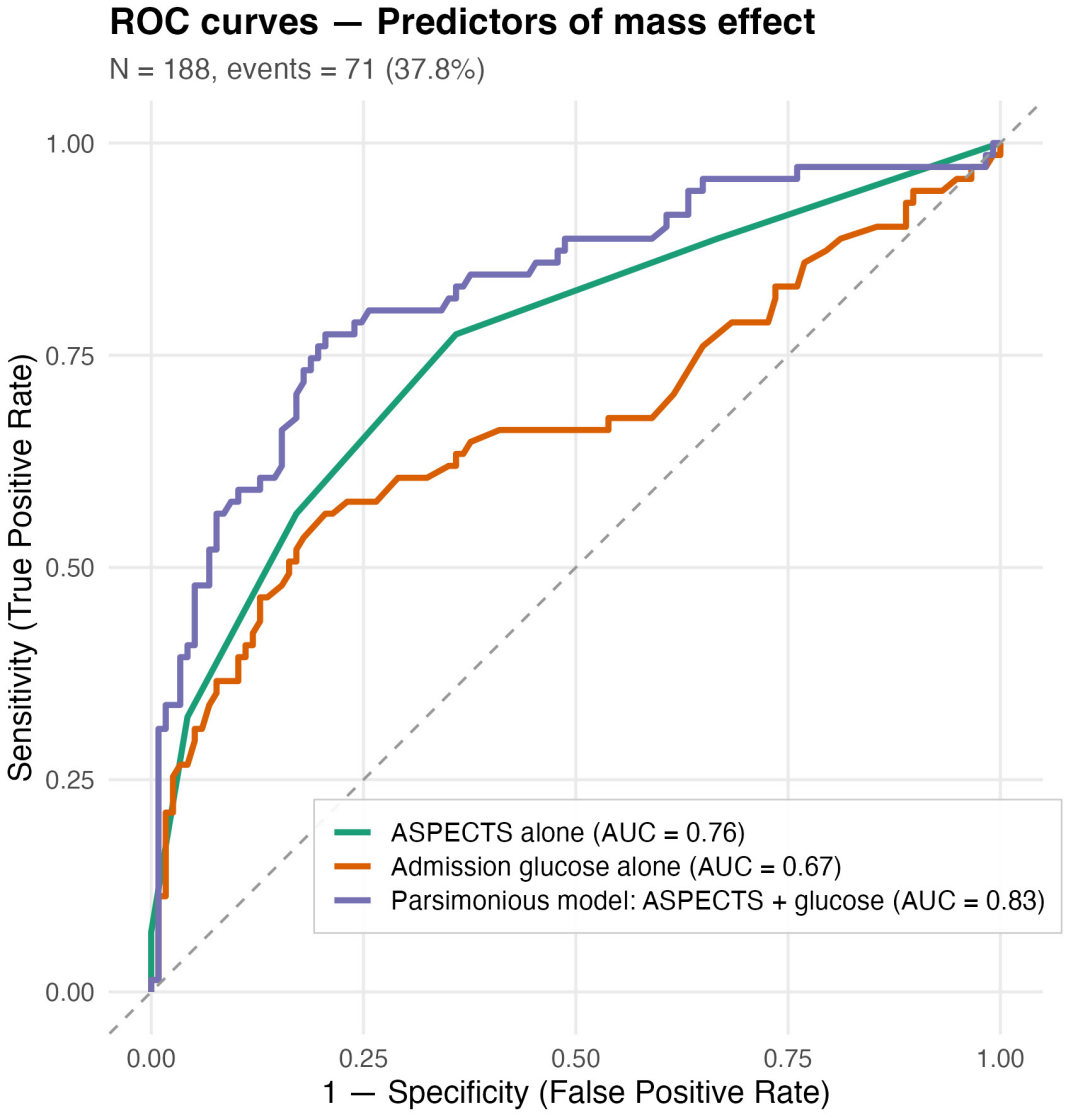
ROC curves for prediction of mass effect. ASPECTS alone, admission glucose alone, and the parsimonious two-variable model (ASPECTS + admission glucose). AUC values shown are apparent; the optimism-corrected AUC of the parsimonious model was 0.82. AUC, area under the curve; ASPECTS, Alberta stroke program early CT score.

### Subgroup analysis

In the subgroup analysis, the treatment effect remained consistent with the primary results, with reduced odds of ME and higher rates of favorable outcome directionally maintained across age, NIHSS, collateral score, and ASPECTS strata. Formal interaction testing was non-significant for all stratifying variables (all *p*-interaction > 0.05), with the single exception of time window for functional outcome (*p*-interaction = 0.028), which was not robust to unweighted sensitivity analysis (*p*-interaction = 0.24). In the ASPECTS 3–5 subgroup, EVT was associated with substantially lower odds of ME (IPTW-aOR: 0.22, 95% CI: 0.08–0.57) and a higher likelihood of achieving mRS 0–3 (IPTW-aOR: 2.75, 95% CI: 1.08–6.98) compared with MM. In the ultra-low ASPECTS (0–2) subgroup, the point estimates were directionally consistent (ME: IPTW-aOR 0.73, 95% CI 0.16–3.39; mRS 0–3: IPTW-aOR 3.49, 95% CI 0.32–37.61) but markedly imprecise and non-significant, and the interaction test was non-significant (*p*-interaction = 0.151 for ME and 0.871 for mRS 0–3) (Figure 5).

**Figure 5:**
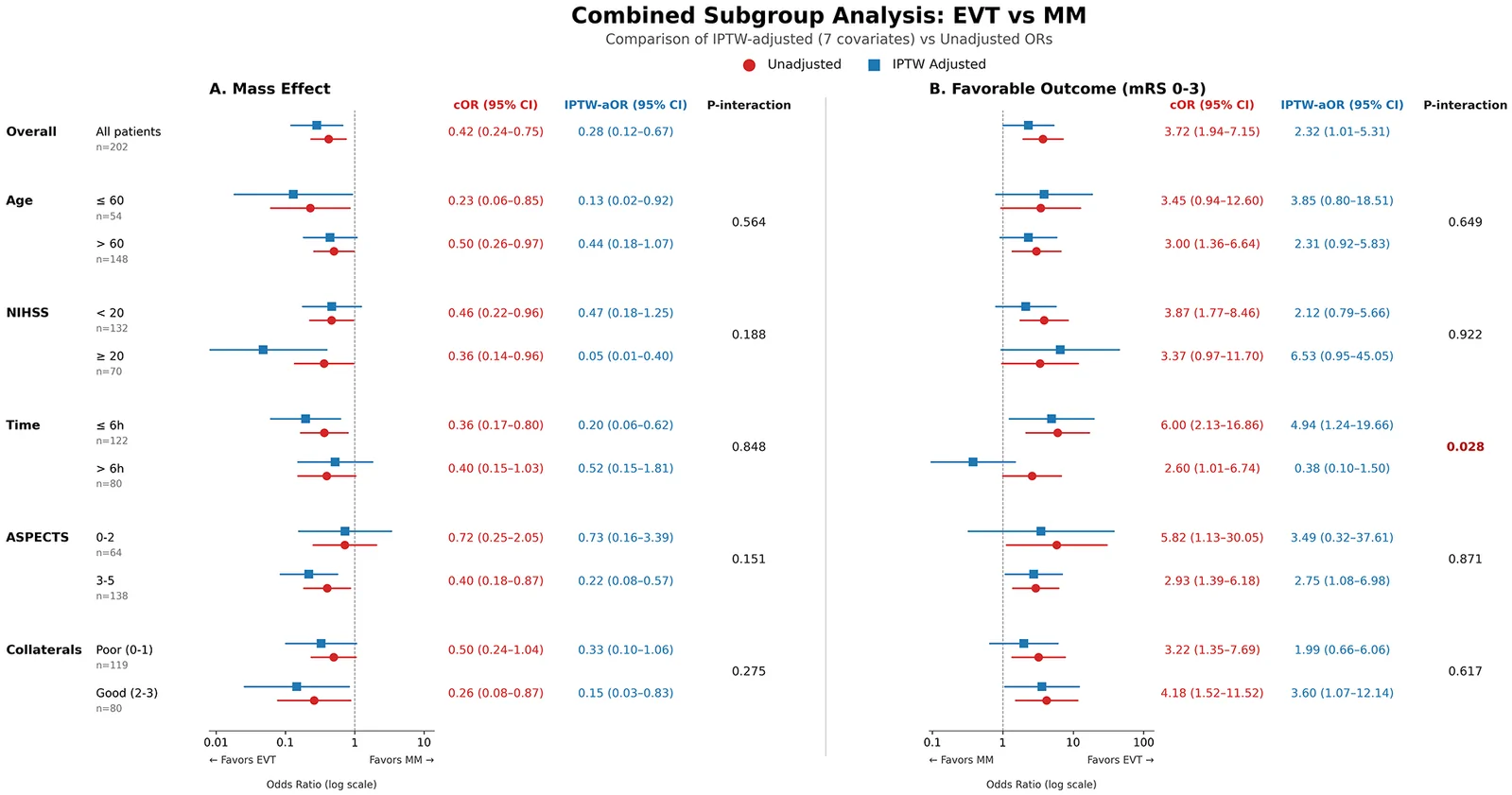
Combined subgroup analysis of the treatment effect on cerebral edema and functional outcome. Forest plots displaying the association between Endovascular Thrombectomy (EVT) and outcomes across prespecified subgroups. **(A)** Risk of Mass Effect (Severe Cerebral Edema). An Odds Ratio (OR) < 1 indicates a benefit of EVT in reducing edema. **(B)** Probability of Favorable Functional Outcome (mRS 0–3) at 90 days. An OR > 1 indicates a benefit of EVT. Estimates are presented for both the unadjusted cohort (red circles) and the Inverse Probability of Treatment Weighting (IPTW) adjusted cohort (blue squares). Horizontal lines represent 95% confidence intervals (CI). EVT, endovascular thrombectomy; MM, medical management; ASPECTS, Alberta stroke program early CT score; NIHSS, National Institutes of Health Stroke Scale.

In the analysis comparing the good and poor reperfusion groups, successful reperfusion was associated with a lower risk of both ME (IPTW-aOR 0.26, 95% CI 0.12–0.57) and MLS (β = −2.0; 95% CI −3.90, −0.11), as well as better functional outcome (IPTW-aOR 3.25, 95% CI 1.54–6.84), compared with unsuccessful reperfusion. Details of the comparison between the two groups are in Table 2.

**Table 2:** Characteristics and outcomes of reperfusion status.

Characteristics of the patients	Good reperfusion (104)	Poor reperfusion (98)	*p*	SMD
**Age, years—Median (IQR)**	66 (60–76)	69 (61–80)	0.733	0.016
**Female sex—%**	46.0%	47.2%	0.876	0.025
**Baseline NIHSS—Median (IQR)**	18 (14–22)	18 (15–20)	0.877	0.042
**Baseline ASPECTS—Median (IQR)**	4 (2–4)	3 (2–5)	0.951	0.026
**Occlusion site—no.(%)**				
ICA	8.2%	9.6%	0.977	0.067
M1 segment of MCA	51.3%	52.2%		
M2 segment of MCA	1.1%	1.4%		
Tandem	39.3%	36.9%		
**Collateral score—Median (IQR)**	1 (1–2)	1 (1–2)	0.481	0.097
**Stroke etiology—no.(%)**				
Large-artery atherosclerosis	43.0%	34.1%	0.339	0.229
Cardioembolic	40.6%	41.7%		
Undetermined or other	16.4%	24.2%		
Median time from onset to admission (IQR) (hours)	4.7 (2.2–9.3)	5.3 (2.5–8.2)	0.660	0.017
≤ 6 h—(%)	59.7%	59.2%	0.949	0.010
>6 h—(%)	40.3%	40.8%		
**Outcomes**	**Good reperfusion (104)**	**Poor reperfusion (98)**	***p***	**IPTW-aOR**
**Primary outcomes**				
Mass effect—% (no.)	27.9% (29)	52.0% (51)	0.001	0.26 (0.12–0.57)
Midline shift (Median–IQR) (mm)	0.0 (0.0–6.2)	5.0 (0.0–10.0)	0.039	β: −2.00 (−3.90, −0.11)
**Secondary outcomes**				
Shift mRS at 90 days—Median (IQR)	3 (2–6)	6 (4–6)	< 0.001	0.36 (0.20–0.64)
mRS 0–3—% (no.)	51.0% (53)	19.4% (19)	< 0.001	3.25 (1.54–6.84)
**Safety outcomes**				
Symptomatic ICH—% (no.)	10.6% (11)	9.2% (9)	0.741	1.58 (0.54–4.62)
Any ICH—% (no.)	58.7% (61)	29.6% (29)	< 0.001	3.09 (1.58–6.04)
Death within 90 days—% (no.)	26.9% (28)	51.0% (50)	< 0.001	0.35 (0.17–0.74)

All effect estimates were adjusted using IPTW with the same propensity-score covariates and doubly-robust outcome-model adjustment for stroke etiology, identical to the primary analysis.

aOR, adjusted odds ratio; ASPECTS, Alberta stroke program early CT score; CI, confidence interval; ICA, internal carotid artery; ICH, intracranial hemorrhage; IPTW, inverse probability of treatment weighting; IQR, interquartile range; M1/M2, first/second segments of the middle cerebral artery; MCA, middle cerebral artery; mRS, modified Rankin Scale; mTICI, modified Thrombolysis in Cerebral Infarction; NIHSS, National Institutes of Health Stroke Scale; SMD, standardized mean difference.

## Discussion

In this prospective, real-world cohort of patients with anterior-circulation large infarct core, EVT was associated with lower odds of clinically significant mass effect, a higher likelihood of a favorable 90-day outcome, and lower mortality, without an excess of symptomatic intracranial hemorrhage. In causal mediation analyses, approximately one-third of the association between EVT and favorable outcome was statistically mediated by attenuation of ME, with the mediated proportion greatest at thresholds capturing severe disability and death—consistent with edema attenuation being a clinically meaningful, though not exclusive, pathway of reperfusion benefit. Importantly, these benefits were evident even when patients were selected using a simplified NCCT-CTA protocol, further supporting the pragmatic utility of this approach in resource-limited settings. ME was independently associated with poor 90-day outcome, whereas lower baseline ASPECTS and higher admission glucose identified patients at greater risk of severe swelling. These findings were consistent across unadjusted and IPTW-adjusted analyses. Although our analyses quantify how much of the observed benefit is statistically mediated by ME, the observational, non-randomized design means that these findings are hypothesis-generating and do not establish the biological mechanisms through which that benefit arises.

Reported relationships between reperfusion and CED differ substantially across studies, and these differences appear to depend heavily on how edema is defined and measured, when it is assessed, and which large-core patients are included. We therefore outline the rationale for our endpoint before comparing our findings with previous work. We defined the primary outcome as ME due to severe CED, operationalized as a maximum MLS of at least 5 mm on follow-up imaging within 72 h. We selected this threshold for three reasons. First, it is clinically actionable: MLS of at least 5 mm is widely used as a trigger for intensified neurological monitoring, osmotherapy, and consideration of decompressive hemicraniectomy, and it has been repeatedly associated with poor functional outcome and mortality (18, 29). Second, it can be measured reliably on routine NCCT without dedicated post-processing software, which is essential for external applicability in settings where quantitative imaging tools are not available. Third, a binary, clinically anchored endpoint is well suited to mediation analysis because it corresponds to the clinical state that treatment decisions actually target.

However, MLS is a late and indirect surrogate of edema with recognized limitations. As Broocks et al. have emphasized, the degree of MLS depends heavily on pre-existing intracranial reserve: older patients with substantial atrophy may accumulate considerable edema without developing significant MLS, whereas younger patients with limited intracranial compartment reserve may exhibit pronounced MLS despite more modest water uptake (30). MLS is also insensitive to earlier and milder stages of edema. Quantitative net water uptake (NWU), derived from densitometric changes on NCCT, directly quantifies tissue water content within the infarct (31). applies across the full range of lesion sizes, including those without visible shift, and is therefore a more sensitive and biologically informative measure of edema evolution (32). NWU, however, requires dedicated post-processing software and standardized densitometric calibration, which are not routinely available in many centers, especially in low- and middle-income countries, where a substantial proportion of the global stroke burden is treated. In these settings, quantitative edema biomarkers remain largely a research tool, whereas MLS is obtained from the same routine follow-up NCCT that already informs bedside decisions. Beyond feasibility alone, a shift of at least 5 mm is itself the clinical event that triggers escalation of care—intensified neurological monitoring, osmotherapy, or referral for decompressive hemicraniectomy—and therefore carries a decisional relevance that a continuous imaging biomarker does not. We regard the two metrics as complementary rather than competing: NWU characterizes the tissue-level process of water accumulation, whereas MLS of at least 5 mm marks its clinically consequential endpoint. Our findings should accordingly be read as addressing the clinically actionable end of this spectrum, and they cannot be assumed to apply to the earlier stages of edema captured by NWU.

Using this threshold-defined endpoint, EVT was associated with a lower incidence of ME in our cohort. This association became clearer when reperfusion quality was examined directly, with good reperfusion showing markedly lower odds of ME and a smaller midline shift compared with poor reperfusion. Notably, this relationship with continuous MLS was evident in the as-treated comparison but not in the intention-to-treat comparison of EVT vs. medical management, consistent with the interpretation that achieved reperfusion, rather than treatment assignment alone, relates most directly to edema formation. Mechanistically, cerebral edema after large-vessel occlusion develops in successive stages that partly overlap in time. In the first hours after occlusion, swelling is driven predominantly by cytotoxic (cellular) edema, in which energy failure and impaired Na^+^/K^+^-ATPase activity redistribute water from the extracellular to the intracellular compartment—the mechanism underlying diffusion restriction on MRI and the rationale for osmotic therapy rather than corticosteroids in ischemic stroke (33). As ionic gradients are established across an initially intact blood–brain barrier, additional water is drawn into the lesion, and only later, with progressive barrier breakdown, does a vasogenic component contribute substantially to lesion growth and mass displacement (30). In partially viable tissue, early recanalization may limit persistent hypoperfusion, reduce infarct expansion, and attenuate the transition from cytotoxic and ionic edema to sustained vasogenic edema, thereby lowering the likelihood of ME; a comparable interpretation has been advanced by Broocks and colleagues, who suggested that restoring perfusion while ionic edema still predominates may interrupt sodium-driven water influx before vasogenic swelling becomes established (30). Consistent with this interpretation, EVT was associated with substantially lower edema progression measured by NWU in the randomized TENSION cohort (4.7% vs. 15.9%) (30), and exploratory analyses from ANGEL-ASPECT showed a more durable reduction in net water uptake after EVT, suggesting reduced water accumulation within the ischemic lesion despite an early increase in MLS (20). At the same time, in severely injured tissue with advanced blood–brain barrier disruption, reperfusion may transiently accentuate capillary leakage and early swelling, which likely may explain why the SELECT2 secondary analysis (21) observed greater continuous MLS after EVT and why the ANGEL-ASPECT secondary analysis (20) demonstrated an early increase in MLS at 24 ± 12 h that was no longer evident at later time points. These apparently discordant findings can be reconciled by differences in endpoint selection, imaging time windows, and exposure definition. Our study focused on clinically relevant ME defined by MLS ≥ 5 mm within 72 h in a real-world cohort, whereas SELECT2 (21) emphasized continuous MLS over 7 days in an advanced imaging-defined large-core population, and ANGEL-ASPECT (20) was particularly informative regarding transient early post-EVT swelling. Furthermore, this interpretation also aligns with the “bimodal response” hypothesis proposed in the HERMES meta-analysis (19), in which reperfusion was not associated with greater MLS overall but did exacerbate edema in patients with very extensive established ischemia (core >130 mL or ASPECTS ≤ 3), while remaining clinically beneficial in moderate-large cores (80–130 ml or ASPECTS 4–5) with salvageable penumbra. In that context, successful reperfusion may help limit the “ghost core” phenomenon described by Ter Schiphorst (34), in which profound hypoperfusion produces early edema-related hypoattenuation on NCCT, despite residual tissue viability. Consistent with this concept, Xu et al. reported that successful recanalization and reperfusion were associated with a lower likelihood of malignant CED, with the edema-mediated pathway mainly present in patients with ASPECTS 3–5 (25), concordant with our subgroup findings and with the ASPECTS 3–5 population enrolled in TENSION (7, 30). Taken together, successful reperfusion may be associated with less severe CED, and thereby with attenuation of the intracranial pressure elevation that can culminate in fatal transtentorial herniation, potentially addressing a major contributor to mortality in this high-risk population.

Beyond its association with mass effect, EVT was associated with better functional recovery in our cohort, including a higher likelihood of achieving mRS 0–3, lower 90-day mortality, and a favorable shift in the overall mRS distribution at 90 days. These findings are consistent with the recent ATLAS individual-patient-data meta-analysis of six randomized trials in large-core stroke, in which EVT improved the 90-day mRS distribution (adjusted generalized odds ratio 1.63, 95% CI 1.42–1.88) and reduced mortality (31.1% vs. 37.3%; adjusted risk ratio 0.82, 95% CI 0.70–0.97) (9). Our favorable outcome rate of 47.1% exceeds the pooled ATLAS estimate for mRS 0–3 (36.5%) (9) and is close to that reported in ANGEL-ASPECT (47.0%) (6), while our 90-day mortality of 30.3% was almost identical to the pooled figure of 31.1% (9). Although our sICH rate of 10.1%, defined by SITS-MOST criteria, appears higher than the pooled ATLAS estimate of 1.1%, the two are not directly comparable: ATLAS applied adjudicated Heidelberg Bleeding Classification criteria centrally, whereas our rate is consistent with those reported in individual trials using clinical definitions, such as RESCUE-Japan LIMIT (9.0%) (4) and LASTE (9.6%) (8), and it was not accompanied by excess mortality. That these outcomes were obtained in patients selected without advanced perfusion imaging supports the pragmatic applicability of an NCCT-CTA pathway. Establishing that EVT is beneficial in this population is, however, no longer the principal question; the more informative issue is through which pathways that benefit operate.

Our mediation analysis addressed this question directly. The proportion of the EVT–outcome association attributable to attenuation of ME was similar in the intention-to-treat (29.5%) and as-treated (29.0%) analyses, with the as-treated estimate yielding a more precisely estimated indirect path. Because the as-treated comparison is internal to thrombectomy-treated patients, it functions as a natural experiment that reduces, although does not eliminate, the influence of treatment-selection bias. Notably, the proportion mediated by ME rose monotonically across mRS thresholds, reaching 41.5% at mRS ≤ 4 in the as-treated analysis, suggesting that prevention of ME is most prominent in averting severe disability and death.

These estimates align closely with mediation analyses performed with different edema metrics in different populations. In the randomized TENSION cohort, approximately 34% of the EVT-related improvement in 90-day mRS was associated with reduced NWU, compared with 14% attributable to change in lesion extent (30); Broocks and colleagues (22) had earlier demonstrated NWU-based mediation in a low-ASPECTS cohort. An analysis from the MAGIC registry by Xu et al. (25) similarly found that incorporating malignant CED attenuated the reperfusion–outcome coefficient by approximately 32.5%, with formal causal mediation reaching statistical significance in the ASPECTS 3–5 subgroup (11.5% mediated; *p* = 0.028) but not in ASPECTS 0–2 patients (25). Importantly, our findings complement an earlier mediation analysis of the HERMES dataset, in which reduction of follow-up infarct volume explained only 12% of EVT's clinical benefit (19). The directional convergence of these methodologically distinct analyses across independent large-core cohorts strengthens the interpretation that ME attenuation is a generalizable rather than cohort-specific pathway of reperfusion benefit, and one that appears to account for a larger share of that benefit than reduction of final infarct extent. Edema attenuation and infarct volume reduction therefore appear to represent distinct and partly independent components of the treatment effect, neither of which accounts for the majority of the benefit; a substantial share evidently operates through further mechanisms, including penumbral salvage, blood–brain barrier preservation, and metabolic restoration. This framework also helps to reconcile an apparent paradox in SELECT2, in which functional outcomes improved with EVT even though rates of cerebral edema and decompressive hemicraniectomy were not reduced;(21) if only about one-third of the benefit operates through edema attenuation, functional benefit could still emerge where an edema signal is not detected, and differences in how and when edema was measured, as discussed above, may further account for its absence.

However, our mediation analysis in a non-randomized cohort rests on identification assumptions that cannot be verified empirically, most importantly the absence of unmeasured confounding of the mediator–outcome relationship; unrecorded differences in the intensity of neurocritical care, for example, could bias the mediated proportion in either direction. In addition, as Broocks and colleagues have noted for their own trial-based estimates, proportions mediated should be regarded as approximate, model-dependent indicators rather than precise causal quantities (30), a caution that applies with greater force to observational data such as ours.

Although our study suggests that EVT is associated with a lower incidence of CED compared to medical treatment, severe swelling with ME remains common in this population. Lower ASPECTS and higher admission glucose independently predicted ME in our cohort, which is biologically plausible and broadly consistent with prior studies linking more advanced ischemic injury, impaired collateral support, and metabolic stress to worse edema evolution (18, 35–37). Importantly, our parsimonious 2-variable model—based on two routinely available admission parameters—could be operationalized at the bedside to identify patients at the highest risk of ME, supporting individualized intensification of neurological monitoring, the timing of repeat imaging, or early escalation to osmotherapy or decompressive hemicraniectomy. Whether pairing EVT with standardized edema-targeted management, including optimized blood glucose and blood pressure control alongside these measures, further improves outcomes remains to be tested prospectively. In subgroup analysis, the non-significant estimate in the ultra-low ASPECTS (0–2) stratum is underpowered and hypothesis-generating, not evidence of effect modification or loss of EVT benefit; its direction is nonetheless consistent with the bimodal pattern discussed above (19, 38). Comparable uncertainty applies to patients with poor collaterals, unsuccessful reperfusion, or late-window treatment, in whom small subgroup sizes limit certainty and underscore the need for future research to clarify EVT's role in CED among these high-risk patients. Further research on cerebral edema—including its causes, mechanisms, and progression—as well as external validation of simple bedside risk models such as ours, remains crucial. Therapeutic goals aimed at preventing or limiting edema progression should also be clarified, particularly to reduce malignant swelling and clinically significant ME. In this regard, adjunctive approaches such as anti-edema agents, including intravenous glibenclamide (39), and emerging neuroprotective agents such as ApTOLL, which has shown encouraging results in reducing infarct growth and edema, particularly in patients with incomplete reperfusion or fast infarct progression (40), may be promising, although efficacy in large-core stroke still requires validation.

### What this study adds

The efficacy of EVT in large-core stroke is now established, and the mediating role of edema has been demonstrated in a randomized cohort using quantitative NWU (30). Against this background, the contribution of the present work lies less in the direction of its findings than in the conditions under which they were obtained. Whereas previous mediation analyses have relied on quantitative imaging biomarkers requiring dedicated post-processing, we show that a comparable mediated proportion can be recovered from a binary endpoint read off routine NCCT at the bedside; the closeness of the two estimates, obtained with different metrics in different populations and study designs, suggests that the edema pathway is not an artifact of how edema is quantified. The as-treated comparison of successful vs. unsuccessful reperfusion provides an internal replication within our own cohort, yielding a nearly identical estimate through a natural experiment less susceptible to treatment-selection bias than the between-group comparison. Our two-variable admission model then extends this from explanation to anticipation, identifying patients at risk of severe swelling using only parameters available in any emergency department.

A further contribution concerns the selection pathway itself. The pivotal trials enrolled patients under defined ASPECTS thresholds and, for the most part, within restricted time windows, and current guidance on extending thrombectomy to patients with very extensive ischemic change or late presentation remains cautious (10). Our consecutive cohort was assembled without an ASPECTS floor and admitted up to 24 h from onset, using only NCCT and CTA. Although the median ASPECTS was 4 and most patients presented in the earlier part of the window, so that experience at the extremes of both criteria remains limited, these data speak to the feasibility of operating such a pathway where perfusion imaging is unavailable—a question of practical consequence as thrombectomy capacity expands in resource-limited regions.

## Limitations

Several limitations should be acknowledged. First, and most importantly, this was a single-center, non-randomized study in which the decision to perform EVT rested with the treating clinicians rather than on allocation. Baseline imbalances—notably larger infarcts and less frequent intravenous thrombolysis in the medically managed group—were addressed with IPTW and doubly robust adjustment. Three covariates nevertheless remained imbalanced after weighting and were not included in the outcome models (Supplementary Section S1.3). Two of them predict worse outcomes and were more frequent in the thrombectomy arm, so residual confounding from them would make the estimated benefit conservative; the third, intravenous thrombolysis, was also more frequent in that arm but predicts better outcomes, and is therefore the one source that would bias the estimate away from the null. Residual and unmeasured confounding by indication cannot be excluded. Residual and unmeasured confounding by indication cannot be excluded. To permit independent appraisal of these analyses, the propensity score specification, standardized mean differences before and after weighting, the distribution of stabilized weights, and positivity checks are presented in Supplementary Material. Second, as discussed above, mediation analysis in this setting rests on identification assumptions that cannot be tested empirically. Third, our edema endpoint is a late and indirect surrogate whose expression depends on intracranial reserve, and we did not measure NWU, so earlier or milder stages of edema were not captured and our estimates cannot be placed on a common scale with NWU-based analyses. Fourth, imaging protocols were not fully standardized: follow-up imaging was obtained with NCCT or MRI at 24 ± 6 h in all patients, whereas repeat imaging within 72 h was performed on clinical grounds, and resource constraints meant that serial imaging near 72 h and through days 5–7 was not uniformly obtained in medically managed patients; later-developing ME may therefore have been under-ascertained in that group, and a robust time-to-event analysis was precluded. Fifth, patients without follow-up imaging at 24 ± 6 h were excluded, which may have influenced the results. Sixth, subgroup analyses—particularly in the ultra-low ASPECTS 0–2 stratum—were underpowered, and all interaction findings should be considered exploratory. Finally, the modest single-center sample limits generalizability, and external validation of both the mediation findings and the risk model is required.

## Conclusion

In this real-world cohort of anterior circulation large-core stroke selected with a simplified NCCT-CTA workflow, EVT was associated with better 90-day functional outcomes and lower odds of mass effect, particularly when successful reperfusion was achieved, without an excess of symptomatic intracranial hemorrhage. Approximately one-third of the observed functional benefit was statistically mediated by attenuation of severe cerebral edema, with this pathway most prominent in averting severe disability and death. Mass effect remained a strong marker of poor prognosis, whereas lower ASPECTS and higher admission glucose identified patients at greater risk of severe swelling. Because these findings derive from a non-randomized single-center cohort, they should be regarded as hypothesis-generating and require confirmation in larger multicenter studies incorporating quantitative edema biomarkers.

## Data Availability

The raw data supporting the conclusions of this article will be made available by the authors, without undue reservation.
