# Supplementary material for: Thrombectomy improves functional outcome in large core stroke partly by preventing severe cerebral edema: a real-world mediation analysis
Source: Front Neurol. 2026 Oct 16;17:1936247. doi: 10.3389/fneur.2026.1936247 (PMC13719343; doi:10.3389/fneur.2026.1936247)
Supplement: Supplementary file 1 [file Supplementary_File_1.docx]

**Thrombectomy Improves Functional Outcome In Large Core Stroke Partly by Preventing Severe Cerebral Edema:**

**A Real-World Mediation Analysis**

These supplementary materials provide

- (S1) Study population: eligibility, baseline characteristics, and weighting diagnostics.
- (S2) Causal mediation analysis – full results
- (S3) Prognostic model for mass effect
- (S4) Medical and surgical management of cerebral edema

**S1. Study population: eligibility criteria, patient flow, baseline characteristics, and weighting diagnostics**

**S1.1 Eligibility criteria**

**General inclusion criteria:**

- Symptomatic patients: Clinical presentation consistent with a diagnosis of acute ischemic stroke.
- Age **≥** 18 years.
- Baseline NIHSS score **≥** 6.
- Neuroimaging criteria:
  - Occlusion of the internal carotid artery (ICA; extracranial or intracranial segments), the M1 segment of the middle cerebral artery (MCA), or the proximal M2 segment of the MCA, confirmed by computed tomography angiography (CTA).
  - Large infarct core definition: ASPECTS ≤ 5 on baseline non-contrast computed tomography (NCCT).
- Time window: Patients with large core stroke admitted within 24 hours of stroke onset (defined as the time the patient was last known well).
- Consent**:** Informed consent was obtained from the patient or their legal representative to participate in the study.

**General exclusion criteria:**

- Patients with severe end-stage medical comorbidities (e.g., severe cardiac or pulmonary disease) or end-stage malignancy.
- History of prior stroke resulting in pre-existing disability (baseline modified Rankin Scale [mRS] ≥ 3).
- Presence of brain tumors, arteriovenous malformations (AVMs), or acute intracranial hemorrhage on neuroimaging.
- Evidence of cerebral herniation, midline shift, or significant mass effect causing ventricular compression on admission CT scan.

**Medical Management Group Selection Criteria (Control Group):**

- Patients meeting the general inclusion and exclusion criteria of the study.
- Patients or legal representatives who declined endovascular intervention.
- Patients who did not meet the eligibility criteria for endovascular thrombectomy according to the participating stroke center protocol.
- Patients with anatomical anomalies or severe aortic arch tortuosity that prevented endovascular access for thrombectomy.

**Intervention group selection criteria (Endovascular Thrombectomy):**

- Patients or legal representatives who consented to endovascular treatment.
- Patients treated with endovascular thrombectomy (EVT) within 24 hours of stroke onset.

**S1.2 Baseline characteristics of the analytic cohort**

Table S1 reports baseline demographic, clinical, imaging, laboratory, and treatment-time characteristics of the 202 patients in the analytic cohort, before and after inverse probability of treatment weighting (IPTW). After weighting, age, sex, baseline NIHSS, baseline ASPECTS and time from onset to admission all achieved standardized mean differences (SMDs) below 0.03, and the largest occlusion-site contrast was 0.124. Five covariates remained above 0.20 and are considered in Section S1.3. Stroke etiology, which was not a propensity-score covariate, showed the largest increase in imbalance after weighting and was therefore included as a doubly-robust covariate in all subsequent outcome models; the weighting diagnostics are presented in Section S1.3.

Table S1: Baseline characteristics of the analytic cohort, before and after IPTW.

| Characteristics of the patients | Overall Cohort | | | | IPTW Cohort | | |
| --- | --- | --- | --- | --- | --- | --- | --- |
|  | All Patients  (202) | EVT  (119) | MM  (83) | SMD | EVT  (119) | MM  (83) | SMD |
| Age, years - Median (IQR) | 68 (60-78) | 65 (59-74) | 71 (64-83) | 0.476 | 66 (60-78) | 69 (59-78) | 0.004 |
| Female Sex - no.(%) | 47.5% (96) | 50.4% (60) | 43.4% (36) | 0.142 | 45.5% | 45.8% | 0.007 |
| Pre-mRS - Median (IQR) | 0 (0-0) | 0 (0-0) | 0 (0-0) | 0.115 | 0 (0-0) | 0 (0-0) | 0.138 |
| Medical History - no.(%)  TIA/Stroke  Hypertension  Atrial Fibrillation  Diabetes mellitus  Dyslipidaemia  Coronary artery disease  Valvular disease  Heart failure  Anticoagulant Agent  Antiplatelet Agent | 15.3% (31)  79.7% (161)  23.3% (47)  12.9% (26)  37.1% (75)  12.4% (25)  12.9 % (26)  15.4% (31)  12.9% (26)  23.8% (48) | 13.4% (16)  75.6% (90)  26.9% (32)  11.8% (14)  39.5% (47)  14.3% (17)  10.9% (13)  19.3% (23)  15.1% (18)  23.5% (28) | 18.1% (15)  85.5% (71)  18.1% (15)  14.5% (12)  33.7% (28)  9.6% (8)  15.7% (13)  9.6% (8)  9.6% (8)  24.1% (20) | 0.127  0.253  0.212  0.080  0.120  0.144  0.140  0.278  0.167  0.013 | 15.4%  78.3%  24.3%  11.3%  39.9%  15.2%  9.1%  17.1%  12.1%  26.7% | 19.7%  83.5%  16.2%  12.6%  34.7%  8.3%  15.4%  8.6%  8.6%  25.9% | 0.119  0.135  0.196  0.041  0.109  0.211  0.186  0.242  0.107  0.020 |
| Baseline NIHSS – Median (IQR) | 18 (15-21) | 17 (14-21) | 18 (16-21) | 0.281 | 18 (14-22) | 18 (15-20) | 0.029 |
| Systolic blood pressure -Median (IQR) | 140 (120-160) | 140 (120-150) | 140 (130-160) | 0.277 | 140 (120-160) | 140 (120-160) | 0.009 |
| Baseline ASPECTS - Median (IQR)  0-2 -no.(%)  3-5 -no.(%) | 3 (2-4)  31.7% (64)  68.3% (138) | 4 (2-5)  25.2% (30)  74.8% (89) | 3 (2-4)  41.0% (34)  59.0% (49) | 0.415  0.340 | 4 (2-4)  27.8%  72.2% | 4 (2-5)  30.8%  69.2% | 0.016  0.065 |
| Occlusion site - no.(%)  ICA  M1 segment of MCA  M2 segment of MCA  Tandem | 9.9% (20)  49.5% (100)  1.5% (3)  39.1% (79) | 6.7% (8)  47.1% (56)  0.8% (1)  45.4% (54) | 14.5% (12)  53.0% (44)  2.4% (2)  30.1% (25) | 0.253  0.119  0.124  0.319 | 8.1%  51.6%  1%  39.2% | 9.1%  56.3%  1.4%  33.3% | 0.031  0.093  0.029  0.124 |
| Collateral score - Median (IQR) | 1 (1-2) | 1 (1-2) | 1 (1-2) | 0.217 | 1 (1-2) | 1 (1-2) | 0.088 |
| Blood tests  Glucose  HGB (g/L)  Creatinine  ALT  LDL | 122.4 (106.5-153.6)  136 (126-148)  72.3 (59.8-89.1)  19.8 (14.7-27.1)  3.2 (2.4-3.9) | 120.6 (106.6-151.2)  137 (126-150)  68.7 (59.2-88.4)  20.5 (14.7-27.8)  3.2 (2.4-3.9) | 127.8 (107.1-160.2)  135 (126-145)  75.1 (62.6-90.4)  19.6 (15.0-26.2)  3.1 (2.4-4.2) | 0.116  0.117  0.233  0.124  0.112 | 121.8 (106.7-151.2)  135 (124-150)  69.9 (59.9-88.7)  18.9 (15.1-27.0)  2.9 (2.2-3.8) | 132.9 (102.1-167.4)  135 (121-143)  74.8 (60.0-88.7)  20.0 (15.8-26.9)  3.1 (2.4-4.2) | 0.154  0.155  0.162  0.114  0.174 |
| Intravenous thrombolysis - no.(%) | 20.3% (41) | 28.6% (34) | 8.4% (7) | 0.537 | 23.6% | 11.2% | 0.330 |
| Stroke Etiology- no.(%)  Large-Artery Atherosclerosis  Cardioembolic  Undetermined or other | 36.1% (73)  42.6% (86)  21.3% (43) | 38.7% (46)  43.7% (52)  17.6% (21) | 32.5% (27)  41.0% (34)  26.5% (22) | 0.128  0.055  0.215 | 45.5%  39.8%  14.7% | 29.7%  39.1%  31.2% | 0.331  0.013  0.400 |
| Onset type  Unknown  Witnessed  Wake-up | 6.9% (14)  65.8 % (133)  27.2% (55) | 5.9% (7)  71.4% (85)  22.7% (27) | 8.4% (7)  57.8% (48)  33.7% (28) | 0.099  0.287  0.247 | 5.1%  62.4%  32.5% | 8.1%  68.1%  23.8% | 0.117  0.121  0.195 |
| Median time from onset to admission (IQR) (hours)  $\boldsymbol{\leq}$ 6 hours – no.(%)  $\boldsymbol{>}$ 6 hours – no. (%) | 4.8 (2.2-9.0)  60.4% (122)  39.6% (80) | 3.5 (1.8-7.1)  70.6% (84)  29.4% (35) | 6.9 (4.2-11.0)  45.8% (38)  54.2% (45) | 0.611  0.520 | 4.8 (2.0-9.5)  60.4%  39.6% | 4.9 (2.5-7.9)  58.1%  41.9% | 0.008  0.048 |
| Median time (IQR) (minute)  Admission to Needle  Admission to puncture | 41.0 (34.5-57.5) | 40.5 (34.8-56.3)  104 (86.5-121.0) | 46.0 (36.5-53.0) | -  - | 40.1 (34.1-54.6)  104 (84.0-121.0) | 49.7 (39.5-54.9)  - | -  - |
| mTICI  0-2a  $\boldsymbol{\geq}$ 2b |  | 12.6% (15)  87.4% (104) |  |  | 11.5%  88.5% |  |  |

*Continuous variables are reported as median (IQR); categorical variables as % (n). The Overall Cohort columns present unweighted statistics; the IPTW Cohort columns present statistics weighted by stabilized propensity-score weights (covariates: age, sex, baseline NIHSS, baseline ASPECTS, time from onset to admission, occlusion site). However, balance did not improve uniformly: stroke etiology, which was not among the propensity-score covariates, showed the largest increase in imbalance after weighting (large-artery atherosclerosis, SMD 0.061 to 0.158). It was therefore explicitly included as a covariate in the final doubly robust multivariable regression models.*

*Abbreviations: ASPECTS, Alberta Stroke Program Early CT Score; EVT, endovascular thrombectomy; ICA, internal carotid artery; IPTW, inverse probability of treatment weighting; IQR, interquartile range; M1/M2, first/second segments of the middle cerebral artery; MCA, middle cerebral artery; MM, medical management; mRS, modified Rankin Scale; mTICI, modified Thrombolysis in Cerebral Infarction; NIHSS, National Institutes of Health Stroke Scale; SMD, standardized mean difference; TIA, transient ischemic attack.*

## **S1.3 Propensity score model and weighting diagnostics**

This section reports the specification of the propensity score model, the distribution of the resulting stabilized weights, the positivity assessment, covariate balance before and after weighting, and the extent of missing data. It is provided so that the weighting procedure underlying every adjusted estimate in the main text can be appraised independently.

### **S1.3.1 Propensity score model**

The propensity score was the estimated probability of receiving endovascular thrombectomy (EVT) rather than medical management (MM), obtained from a multivariable logistic regression model containing six covariates specified in advance on clinical grounds as determinants of both treatment selection and outcome: age, sex, baseline NIHSS, baseline ASPECTS, time from onset to admission, and occlusion site (Table S1A). All six were recorded for every patient, so the model was fitted on the complete cohort of 202 patients and no imputation was required to derive the weights.

Three covariates were independently associated with the treatment decision: each additional year of age reduced the odds of EVT by 5% (OR 0.95, P < 0.001), each additional ASPECTS point increased them by 48% (OR 1.48, P = 0.002), and each additional hour from onset to admission reduced them by 14% (OR 0.86, P < 0.001). This is the expected signature of confounding by indication in a setting where thrombectomy is offered selectively to younger patients with smaller infarcts who present earlier. Model fit was moderate (null deviance 273.58 on 201 degrees of freedom; residual deviance 221.03 on 193; AIC 239.03), which is the desirable property for a propensity score: a model that separated the two arms almost perfectly would indicate a violation of positivity rather than a good fit.

**Table S1A.** Propensity score model for receipt of endovascular thrombectomy (n = 202).

| **Term** | **β** | **SE** | **OR** | **P** |
| --- | --- | --- | --- | --- |
| Intercept | 4.170 | 1.516 | 64.73 | 0.006 |
| Age, per year | −0.054 | 0.015 | 0.95 | <0.001 |
| Male sex (vs female) | −0.519 | 0.349 | 0.59 | 0.137 |
| Baseline NIHSS, per point | −0.039 | 0.037 | 0.96 | 0.285 |
| Baseline ASPECTS, per point | 0.393 | 0.125 | 1.48 | 0.002 |
| Time from onset to admission, per hour | −0.153 | 0.040 | 0.86 | <0.001 |
| Occlusion site: M1 MCA (vs ICA) | 0.376 | 0.564 | 1.46 | 0.505 |
| Occlusion site: M2 MCA (vs ICA) | −1.082 | 1.419 | 0.34 | 0.445 |
| Occlusion site: tandem (vs ICA) | 1.121 | 0.598 | 3.07 | 0.061 |

Multivariable logistic regression with EVT (vs medical management) as the dependent variable. β, regression coefficient on the log-odds scale; SE, standard error; OR, odds ratio (exponentiated β). Reference categories: female sex; ICA occlusion. Null deviance 273.58 (201 df); residual deviance 221.03 (193 df); AIC 239.03.

### **S1.3.2 Distribution of stabilized weights**

Stabilized inverse-probability weights were derived from the fitted propensity scores (Table S1B). The distribution was centred close to unity (mean 0.992, median 0.803, interquartile range 0.665–1.065), indicating that the weighted pseudo-population was approximately the same size as the observed cohort and that the weighting did not artificially inflate the effective sample. The distribution was right-skewed at its upper extreme: the 99th percentile was 3.486 and the single largest weight was 5.902. Weights were not truncated or trimmed, so that the reported estimates reflect the observed data rather than a modified version of it; no individual weight approached the threshold of 10 that is conventionally taken to signal an unduly influential observation. Robust (sandwich) variance estimators were used in all weighted models to account for the weighted design.

**Table S1B.** Distribution of stabilized inverse-probability-of-treatment weights.

| **Statistic** | **Value** |
| --- | --- |
| Number of patients | 202 |
| Mean | 0.992 |
| Standard deviation | 0.654 |
| Minimum | 0.422 |
| 1st percentile | 0.441 |
| 1st quartile | 0.665 |
| Median | 0.803 |
| 3rd quartile | 1.065 |
| 99th percentile | 3.486 |
| Maximum | 5.902 |

Stabilized weights were calculated as the marginal probability of the treatment actually received divided by the propensity score for that treatment. No truncation or trimming was applied.

### **S1.3.3 Positivity and common support**

Positivity was assessed by comparing the range of propensity scores observed in the two arms (Table S1C). Scores ranged from 0.027 to 0.887 under medical management and from 0.100 to 0.975 after thrombectomy, giving a common support region of 0.100 to 0.887 within which both treatments were observed. Overlap was therefore substantial across most of the score distribution, but it was not complete at the extremes: a small number of medically managed patients had propensity scores below the lowest score observed in the EVT arm, and a small number of EVT-treated patients had scores above the highest observed under medical management. For those patients the alternative treatment was, in practice, never chosen, and the weighted estimates in those regions rest on model-based extrapolation rather than on observed counterfactuals. We report the full range rather than trimming the cohort to common support, and regard the non-overlapping tails as a boundary on the generalizability of the weighted estimates rather than as a correctable feature of the data.

**Table S1C.** Propensity score range by treatment arm (positivity assessment).

| **Group** | **Minimum** | **Maximum** |
| --- | --- | --- |
| Medical management (n = 83) | 0.027 | 0.887 |
| Endovascular thrombectomy (n = 119) | 0.100 | 0.975 |
| Common support region | 0.100 | 0.887 |

The common support region is the interval within which propensity scores were observed under both treatments.

### **S1.3.4 Covariate balance before and after weighting**

Standardized mean differences were computed with the cobalt package in R using the average-treatment-effect estimand and the pooled standard deviation of the unweighted sample as the denominator, for continuous and for categorical covariates alike, with multi-level variables expressed as one contrast per category. This is the convention to which the conventional thresholds of 0.10 and 0.20 refer. Table 2 uses the same standardization, computed with the tableone package; the only difference is that tableone summarizes a multi-level variable with a single value across all categories rather than one contrast per category. Admission-to-needle and admission-to-puncture times are process-of-care measures recorded after admission rather than baseline covariates and are defined only in subgroups; standardized mean differences are therefore not reported for them.

After weighting, the five non-imaging propensity-score covariates all fell below 0.03 and the largest occlusion-site contrast was 0.124. Fifteen of the 36 covariate contrasts fell below 0.10, and 16 lay between 0.10 and 0.20. Balance did not improve uniformly, however: for a small number of covariates outside the propensity score model, the standardized mean difference was larger after weighting than before. This is expected, since weighting targets only the covariates entered into the model.

Five covariates exceeded 0.20 after weighting: the two stroke-etiology categories (large-artery atherosclerosis 0.128 to 0.331; undetermined or other 0.215 to 0.400), intravenous thrombolysis (0.537 to 0.330), heart failure (0.278 to 0.242) and coronary artery disease (0.144 to 0.211). The last two illustrate the sensitivity of the standardized scale to prevalence: coronary artery disease differs between the weighted arms by 6.9 percentage points (15.2% versus 8.3%), a difference that is modest in absolute terms but is divided by a small pooled standard deviation for a covariate of this prevalence.

Stroke etiology showed the largest increase in imbalance after weighting and was carried into every outcome model as a doubly-robust adjustment. The remaining three were not, for three reasons. First, the doubly-robust models already contain ten degrees of freedom against 80 mass-effect events and 72 favorable-outcome events, approximately eight and seven events per variable; adding three further terms would reduce these to approximately six and five, below the range in which logistic regression estimates remain stable. Second, cardiac comorbidity is closely correlated with cardioembolic etiology, which is already in the models, so much of this information is captured indirectly. Third, the direction of any residual confounding differs across these covariates: heart failure and coronary artery disease were both more frequent in the thrombectomy arm, and both predict worse outcome, so residual imbalance in them would make the estimated benefit of EVT conservative, whereas intravenous thrombolysis was more frequent in the thrombectomy arm and predicts better outcome and is therefore the one covariate whose residual imbalance would bias the estimate away from the null. This is stated in the Limitations of the main text.

### **S1.3.5 Missing data**

Missing data were confined to laboratory variables and the CTA collateral score, none of which entered the propensity score model (Table S1D). The largest proportion missing was 6.9% for ALT; glucose and LDL cholesterol were each missing in 5.4%, creatinine and collateral score in 1.5%, and haemoglobin in none. Because the six weighting covariates were complete for all 202 patients, the propensity score model and the resulting weights were estimated on the full cohort and no imputation was used in the primary analysis. Patients with missing values for a given laboratory variable contributed to every analysis except the descriptive summary of that variable and its balance statistic.

**Table S1D.** Missing data among variables not included in the propensity score model.

| **Variable** | **n missing** | **% missing** |
| --- | --- | --- |
| Admission glucose | 11 | 5.4 |
| Haemoglobin | 0 | 0.0 |
| Creatinine | 3 | 1.5 |
| ALT | 14 | 6.9 |
| LDL cholesterol | 11 | 5.4 |
| CTA collateral score | 3 | 1.5 |

The denominator is the full analytic cohort of 202 patients. Age, sex, baseline NIHSS, baseline ASPECTS, time from onset to admission, and occlusion site — the six covariates used to estimate the propensity score — were complete for all patients.

**S2. Causal mediation analysis — full results**

**S2.1 Conceptual framework**

We performed causal mediation analysis under the counterfactual framework of Imai, Keele, and Tingley (Psychological Methods, 2010), implemented in the R package mediation (v4.5). Two parallel pre-specified analyses were conducted: (i) an intention-to-treat (ITT) formulation comparing endovascular thrombectomy (EVT) versus medical management (MM); and (ii) an as-treated formulation comparing patients with successful reperfusion (mTICI ≥ 2b) with those with poor reperfusion (mTICI < 2b in the EVT arm plus all medically managed patients).

**S2.2 Variable definitions and adjustment**

The exposure X was binary (EVT = 1 / MM = 0 for ITT; good reperfusion = 1 / poor reperfusion = 0 for as-treated). The mediator M was the binary occurrence of mass effect (midline shift ≥ 5 mm on follow-up imaging within 72 hours). The outcome Y was a binary indicator of mRS ≤ k at 90 days, evaluated at three pre-specified cut-points (k = 2, 3, and 4). Mediator and outcome models were both logistic regressions adjusted for the same six covariates as the propensity score model (age, sex, baseline NIHSS, baseline ASPECTS, time from onset to admission, occlusion site), with stroke etiology retained as an additional covariate. Both analyses were performed on the complete analytic cohort of 202 patients, with no exclusions for missing covariate data. Inverse probability weights were not applied to the mediation models, because the nonparametric bootstrap used for the mediation confidence intervals does not propagate the uncertainty of weight estimation; confounding control in the mediation analysis therefore rests on regression adjustment, and the resulting estimates are covariate-adjusted rather than weighted. The total effect of X on Y was decomposed into the average causal mediation effect (ACME, indirect path through M) and the average direct effect (ADE), with the proportion mediated computed as ACME / (ACME + ADE). Point estimates and 95% confidence intervals were obtained from 1,000 nonparametric bootstrap replicates using the percentile method.

**S2.3 Sensitivity across mRS thresholds**

At the primary endpoint (mRS ≤ 3, favorable outcome), both analyses converged on approximately 30% mediation by mass effect (ITT 29.5%, as-treated 29.0%). The proportion mediated rose monotonically with threshold laxity, reaching 41.5% at mRS ≤ 4 in the as-treated analysis, consistent with the interpretation that mass effect prevention is most clinically prominent in averting severe disability and death (Table S2). The as-treated analysis yielded narrower bootstrap confidence intervals than the ITT analysis at every threshold, reflecting the more proximal causal placement of reperfusion relative to functional outcome.

**Table S2. Sensitivity of mediation estimates across mRS thresholds.**

| **mRS cut-point** | **ACME** | **ADE (95% CI)** | **Total (95% CI)** | **% Mediated (95% CI)** | **P (ACME)** |
| --- | --- | --- | --- | --- | --- |
| **Panel A. EVT vs Medical Management (Intention to treat)** | | | | | |
| mRS ≤ 2 (excellent) | 0.023 | 0.096 (−0.022, 0.187) | 0.119 (0.007, 0.220) | 19.5% (−17.0, 140.4) | 0.082 |
| mRS ≤ 3 (favorable, primary) | 0.044 | 0.104 (−0.028, 0.239) | 0.148 (0.003, 0.293) | 29.5% (−16.7, 161.2) | 0.068 |
| mRS ≤ 4 (functional survival) | 0.066 | 0.020 (−0.111, 0.153) | 0.085 (−0.065, 0.236) | 77.0% (−304.1, 543.1) | 0.072 |
| **Panel B. Good vs Poor Reperfusion (as-treated)** | | | | | |
| mRS ≤ 2 (excellent) | 0.034 | 0.100 (−0.004, 0.194) | 0.134 (0.035, 0.238) | 25.2% (5.4, 105.3) | 0.014 |
| mRS ≤ 3 (favorable, primary) | 0.060 | 0.147 (0.017, 0.281) | 0.206 (0.068, 0.353) | 29.0% (6.7, 73.9) | 0.014 |
| mRS ≤ 4 (functional survival) | 0.093 | 0.130 (−0.007, 0.261) | 0.223 (0.072, 0.355) | 41.5% (13.2, 106.2) | 0.012 |

*All effects on the probability scale. ACME = average causal mediation effect (indirect effect via mass effect); ADE = average direct effect; total = total effect. Covariate adjustment and bootstrap specification are identical to the mediation analysis described in Section S2.2; these models are covariate-adjusted and unweighted. Bootstrap confidence intervals are derived from 1,000 nonparametric percentile replicates.*

**S3. Prognostic model for mass effect**

The main text reports the parsimonious two-variable model (ASPECTS + admission glucose) together with its bootstrap-corrected performance. This section provides supporting analyses that did not fit in the main text: the full ten-variable multivariable model, the rationale for eliminating the CTA collateral score, and the head-to-head comparison of full versus parsimonious models that justifies the parsimonious choice.

**S3.1 Candidate predictors**

Of 202 patients, 188 had complete data for all candidates and were included in the prognostic analysis (71 events; 37.8%). Ten predictors were pre-specified on biological grounds: age, female sex, admission NIHSS, baseline ASPECTS, CTA collateral score (CsCTA), time from onset to admission, admission plasma glucose, occlusion location (ICA / M1 / M2 / tandem), stroke etiology (TOAST), and treatment assignment (EVT vs. medical management). A full multivariable logistic regression was fit; a parsimonious model was obtained by backward AIC selection; both were internally validated by a 500-iteration nonparametric bootstrap.

**S3.2 Full ten-variable multivariable model**

In the full model, three predictors remained independently associated with mass effect (Table S3A): lower ASPECTS, lower CsCTA, and higher admission glucose. The NIHSS and treatment-effect terms attenuated to the null after adjustment, consistent with their influence being mediated through baseline infarct burden and collateral status rather than acting as independent direct contributors.

**Table S3A. Full ten-variable multivariable logistic regression for mass effect (N = 188; events = 71).**

| **Term** | **aOR (95% CI)** | **β** | **SE** | **P** |
| --- | --- | --- | --- | --- |
| Intercept | 2.79 (0.07–112.72) | 1.025 | 1.888 | 0.587 |
| Age | 1.01 (0.97–1.04) | 0.006 | 0.017 | 0.748 |
| Female sex | 1.14 (0.47–2.75) | 0.133 | 0.448 | 0.766 |
| Baseline NIHSS | 0.99 (0.91–1.08) | −0.010 | 0.042 | 0.820 |
| Baseline ASPECTS | 0.51 (0.37–0.70) | −0.679 | 0.162 | <0.001 |
| CTA collateral score | 0.37 (0.20–0.69) | −0.987 | 0.317 | 0.002 |
| Time from onset to admission  (per hour) | 1.02 (0.93–1.12) | 0.017 | 0.048 | 0.716 |
| Admission glucose (per mg/dL) | 1.02 (1.01–1.02) | 0.016 | 0.004 | <0.001 |
| Occlusion site: M1 MCA (vs ICA) | 0.37 (0.10–1.35) | −0.986 | 0.658 | 0.134 |
| Occlusion site: tandem (vs ICA) | 0.68 (0.17–2.69) | −0.382 | 0.700 | 0.586 |
| Occlusion site: M2 MCA (vs ICA) | 1.18 (0.07–18.61) | 0.165 | 1.407 | 0.906 |
| Aetiology: cardioembolic  (vs large-artery atherosclerosis) | 1.40 (0.55–3.56) | 0.336 | 0.476 | 0.480 |
| Aetiology: undetermined or other  (vs large-artery atherosclerosis) | 0.75 (0.22–2.50) | −0.294 | 0.618 | 0.634 |
| Treatment: EVT  (vs medical management) | 0.68 (0.27–1.72) | −0.379 | 0.469 | 0.419 |

*aOR, adjusted odds ratio; CI, confidence interval; β, regression coefficient on the log-odds scale; SE, standard error.*

**S3.3 Parsimonious model, elimination of the collateral score, and bootstrap-corrected performance**

The CTA collateral score was eliminated by AIC selection despite its statistical significance in the full model because its prognostic information overlapped substantially with that of ASPECTS once both imaging variables were considered jointly: ASPECTS quantifies the radiographic extent of established early ischemic injury, which is itself a downstream consequence of the same collateral failure that CsCTA measures upstream. Including both variables therefore introduced redundancy without improving bootstrap-corrected discrimination, and AIC penalized the additional parameter accordingly.

On 500-iteration nonparametric bootstrap, the parsimonious model retained almost all of its apparent performance after optimism correction (apparent AUC 0.827 vs bootstrap-corrected 0.821; optimism 0.006), whereas the full ten-variable model showed substantial overfitting (apparent AUC 0.864 vs bootstrap-corrected 0.816; optimism 0.048). The parsimonious model is therefore preferred for bedside use. The final parsimonious coefficients and their bootstrap-corrected performance are shown in Table S3B (Panels A and B); ROC curves are presented in Figure 4.

**Table S3B. Final parsimonious prognostic model (N = 188; events = 71).**

***Panel A. Parsimonious model (backward AIC selection)***

| **Predictor** | **aOR (95% CI)** | **β** | **SE** | **P** |
| --- | --- | --- | --- | --- |
| Intercept | 0.79 (0.22–2.85) | −0.239 | 0.656 | 0.716 |
| ASPECTS (per 1 point) | 0.45 (0.34–0.59) | −0.799 | 0.140 | <0.001 |
| Admission glucose (per mg/dL) | 1.02 (1.01–1.02) | 0.016 | 0.004 | <0.001 |

***Panel B. Model performance (500-iteration bootstrap)***

| **Metric** | **Apparent** | **Bootstrap-corrected (500 iter.)** |
| --- | --- | --- |
| AUC (95% CI) | 0.827 (0.763–0.891) | 0.821 |
| Brier score | 0.156 | 0.162 |
| *Hosmer–Lemeshow goodness-of-fit (g = 10): χ² = 8.21, P = 0.41* | | |

*aOR, adjusted odds ratio; AUC, area under the ROC curve; CI, confidence interval; SE, standard error.*

**S4. Medical and surgical management of cerebral edema**

**S4.1 Institutional eligibility for decompressive hemicraniectomy**

At the participating center, the decision to perform DHC was made after multidisciplinary discussion between the stroke neurologist and neurosurgical team, together with the patient’s family, with the relatives’ preference playing an important role. The clinical criteria were generally based on those used in the HAMLET trial, with one practical extension: age range; DHC was considered only when the surgical and intensive-care teams judged that meaningful functional recovery was plausible:

Inclusion Criteria:

-       Time from stroke onset to treatment ≤ 96 hours

-       Age range: 18-80 years (extended from the original HAMLET 18–60 to reflect real-world practice).

-       NIHSS ≥ 16 for right-sided lesions or ≥ 21 for left-sided lesions.

-       GCS ≤ 13 for right-sided lesions or GCS ≤ 9 for left-sided lesions.

-       Large hemispheric infarction (≥ 2/3 MCA territory) and severe cerebral edema with mass effect (midline shift ≥5 mm)

Exclusion criteria:

- Ischaemic stroke of the whole cerebral hemisphere (ACA, MCA, and PCA territories) or bilateral ischaemic strokes.

**-** Terminal illness with **life expectancy <1 year**

- Known systemic bleeding disorder

- Both pupils were fixed and dilated

- Decreased consciousness is explained partly by **non-edema causes** (e.g., metabolic disturbances or medication)

- Prestroke mRS ≥ 3

- GCS < 5.

Eligibility was framework-based; the binding rate-limiting factors for surgery in our setting were practical and structural rather than purely clinical. The study setting is a lower-middle-income country (LMIC); out-of-pocket treatment costs are high, and many families decline DHC because the cost of EVT itself has already imposed a major financial burden. The supply of neurosurgeons is limited and overloaded relative to acute-stroke volume, further constraining the population that can be operated on within a clinically useful time window. As a consequence, the observed DHC rate in this cohort should be interpreted in this LMIC real-world context rather than against high-income trial settings.

**S4.2 Standard medical management of cerebral edema**

All patients with LIC in this cohort received protocolized supportive measures to limit cerebral edema and intracranial pressure. The institutional protocol comprised:

- **Osmotherapy:** hypertonic saline (3% bolus, with 23.4% reserved for refractory cases) or mannitol 0.5–1 g/kg, initiated for clinical or radiographic deterioration suggestive of raised intracranial pressure.
- **Head-of-bed elevation** to 30° in all patients with suspected raised intracranial pressure.
- **Targeted normocapnia** (PaCO₂ 35–40 mmHg); transient mild hyperventilation only as a temporizing measure for impending herniation.
- **Blood pressure control** within guideline targets: pre-IV-thrombolysis SBP < 185 mmHg and DBP < 110 mmHg; pre-EVT (without IV thrombolysis) BP ≤ 185/110 mmHg; after IV thrombolysis BP < 180/105 mmHg for at least 24 hours; after EVT BP ≤ 180/105 mmHg during and for 24 hours after the procedure; in patients managed without reperfusion therapy, no active BP lowering during the first 48–72 hours when BP was < 220/120 mmHg in the absence of an urgent comorbid indication.
- **Glucose control**: target 140–180 mg/dL; hypoglycemia <60 mg/dL was corrected; intensive control to 80–130 mg/dL was not used.
- **Temperature control:** targeted normothermia, with antipyretics and surface cooling for temperatures > 37.5 °C; therapeutic hypothermia is not routinely indicated.
- **Sodium target** (serum sodium 145–155 mmol/L during osmotherapy).
- **Sedation and analgesia were** titrated to suppress agitation and reduce cerebral metabolic demand in mechanically ventilated patients.
- **Decompressive hemicraniectomy** as detailed in Section S4.1.

**S4.3 Characteristics and 90-day outcomes of the DHC subgroup**

Decompressive hemicraniectomy (DHC) was performed in 16 of 202 patients overall (7.9%): 9 of 119 in the EVT arm (7.6%) and 7 of 83 in the MM arm (8.4%) (P = 1.00). DHC within 48 hours: 12 patients (75%), within 72h 15 patients (93.75%). Among the 80 patients who developed mass effect (37 EVT + 43 MM), DHC was performed in 16 (20%); 64 patients (80%) with mass effect did not undergo surgery, predominantly due to LMIC resource and cost constraints described in Section S4.1.

Baseline severity and imaging burden in the DHC subgroup were similar between treatment arms: median baseline ASPECTS was 2 in both arms, and the maximum midline shift on follow-up imaging was a median of 15.0 mm in both groups (P = 0.914), confirming that surgical patients were comparably affected at the time of decompression regardless of whether they had received EVT.

**Table S4. Characteristics and 90-day outcomes of the decompressive-hemicraniectomy subgroup.**

| **Variable** | **All DHC (n = 16)** | **EVT–DHC (n = 9)** | **MM–DHC (n = 7)** | **P** |
| --- | --- | --- | --- | --- |
| Age, years — median (IQR) | 61.5 (58.0–70.0) | 60.0 (55.0–70.0) | 62.0 (60.5–67.0) | 0.596 |
| Female sex — % (n) | 50.0% (8) | 55.6% (5) | 42.9% (3) | 1.000 |
| Baseline NIHSS — median (IQR) | 18.0 (14.8–24.2) | 16.0 (12.0–21.0) | 20.0 (18.0–25.0) | 0.138 |
| Baseline ASPECTS — median (IQR) | 2.0 (2.0–3.0) | 2.0 (2.0–3.0) | 2.0 (2.0–2.5) | 0.565 |
| Admission glucose, mg/dL — median (IQR) | 162.0 (119.1–255.6) | 122.8 (112.5–295.7) | 163.8 (145.8–219.1) | 0.768 |
| Time from onset to DHC, h — median (IQR) | 40.2 (30.1–50.0) | 33.0 (29.0–40.5) | 47.5 (39.1–59.2) | 0.252 |
| Maximum midline shift, mm — median (IQR) | 15.0 (9.0–16.0) | 15.0 (9.0–16.0) | 15.0 (9.5–15.5) | 0.914 |
| Mass effect (MLS ≥ 5 mm) — % (n) | 100.0% (16) | 100.0% (9) | 100.0% (7) | 1.000 |
| Early neurological deterioration — % (n) | 87.5% (14) | 88.9% (8) | 85.7% (6) | 1.000 |
| 90-day favorable outcome (mRS 0–3) — % (n) | 12.5% (2) | 22.2% (2) | 0.0% (0) | 0.475 |
| 90-day mortality — % (n) | 18.8% (3) | 11.1% (1) | 28.6% (2) | 0.550 |

*Continuous variables are reported as median (IQR); categorical variables as % (n). Between-group comparisons used the Mann–Whitney U test for continuous variables and Fisher's exact test for categorical variables. Time-to-DHC is reported relative to stroke onset. The denominator for "All DHC" is 16 (16/202 of the analytic cohort, 7.9%; 16/80 of the mass-effect subgroup, 20%). Eligibility followed the HAMLET criteria with an age extension as detailed in Section S4.1. Abbreviations: ASPECTS, Alberta Stroke Program Early CT Score; DHC, decompressive hemicraniectomy; END, early neurological deterioration; EVT, endovascular thrombectomy; IQR, interquartile range; MLS, midline shift; MM, medical management; mRS, modified Rankin Scale; NIHSS, National Institutes of Health Stroke Scale.*
